# Network Analysis-Based Approach for Exploring the Potential Diagnostic Biomarkers of Acute Myocardial Infarction

**DOI:** 10.3389/fphys.2016.00615

**Published:** 2016-12-09

**Authors:** Jiaqi Chen, Ling Yu, Siwei Zhang, Xia Chen

**Affiliations:** ^1^Department of Pharmacology, College of Basic Medical Sciences, Jilin UniversityChangchun, China; ^2^Department of Pharmacy, The Second Hospital of Jilin UniversityChangchun, China

**Keywords:** acute myocardial infarction, biomarkers, inflammation, systems biology, hub genes

## Abstract

Acute myocardial infarction (AMI) is a severe cardiovascular disease that is a serious threat to human life. However, the specific diagnostic biomarkers have not been fully clarified and candidate regulatory targets for AMI have not been identified. In order to explore the potential diagnostic biomarkers and possible regulatory targets of AMI, we used a network analysis-based approach to analyze microarray expression profiling of peripheral blood in patients with AMI. The significant differentially-expressed genes (DEGs) were screened by Limma and constructed a gene function regulatory network (GO-Tree) to obtain the inherent affiliation of significant function terms. The pathway action network was constructed, and the signal transfer relationship between pathway terms was mined in order to investigate the impact of core pathway terms in AMI. Subsequently, constructed the transcription regulatory network of DEGs. Weighted gene co-expression network analysis (WGCNA) was employed to identify significantly altered gene modules and hub genes in two groups. Subsequently, the transcription regulation network of DEGs was constructed. We found that specific gene modules may provide a better insight into the potential diagnostic biomarkers of AMI. Our findings revealed and verified that *NCF4, AQP9, NFIL3, DYSF, GZMA, TBX21, PRF1* and *PTGDR* genes by RT-qPCR. *TBX21* and *PRF1* may be potential candidates for diagnostic biomarker and possible regulatory targets in AMI.

## Introduction

Acute myocardial infarction (AMI) is a severe cardiovascular disease that is a serious threat to human life (Roger et al., 2012). AMI can cause congestive heart failure and malignant arrhythmia leading to a high morbidity and mortality (Jameel and Zhang, 2009; Mozaffarian et al., 2016). While thrombolysis and percutaneous coronary intervention (PCI) can significantly improve the prognosis of patients with AMI, there are still many patients with AMI that eventually develops into heart failure or arrhythmia due to the unclear etiology of AMI (Eapen et al., 2012) Looking for the potential diagnostic biomarkers and possible regulatory targets of AMI may help to reduce the mortality of AMI. At present, the systems biology analysis of gene expression profiling provides a better method to elucidate the possible mechanisms of myocardial infarction from the perspective of gene regulation. Using gene expression profiles, we can obtain adequate information about altered gene expression correlating with disease. Kiliszek et al. (2012) utilized a microarray approach to demonstrate that, during ST-segment elevation myocardial infarction (STEMI), many genes have altered expression, including those involved in various pathways related to platelet function, lipid/glucose metabolism and atherosclerotic plaque stability. Based on the system level of gene expression profiling, gene co-expression network analyses could be an alternative method for analyzing expression profiling data (Stuart et al., 2003), in order to gain an insight into molecular regulatory mechanisms of heart diseases caused by myocardial infarction. Gene ontology (GO) and pathway enrichment analyses were used in our study to explore the molecular mechanisms of heart disease induced by myocardial infarction.

Weighted gene co-expression network analysis (WGCNA) was also used in this study, and is generally utilized for illuminating the changes of transcriptome expression patterns in complex diseases (Zhang and Horvath, 2005; Chen et al., 2008; Voineagu et al., 2011). Compared with the standardized analysis of DEGs, the purpose of which is to detect disease-related individual genes, WGCNA aims to recognize higher-order correlation between gene products (Oldham et al., 2008). Moreover, the algorithm of WGCNA can substantially simplify the multiple testing problems that are unavoidable in standard gene-centric methods of microarray expression profiling data analysis; therefore, it is a powerful systematic analysis method that focuses on the coherence function of network modules (Zhao et al., 2010).

Using the bioinformatics methods above, we analyzed GO-tree, pathway action network and gene module alteration in patients with AMI to explore the potential diagnostic biomarkers and possible regulatory targets of AMI in our study. The gene co-expression network was constructed based on the gene expression profiling, and gene modules of peripheral blood were also detected by the WGCNA when AMI occurred. Furthermore, hub genes were recognized, which could be used as biomarkers for assessing the severity of heart diseases and served as promising therapeutic targets for promoting novel therapeutic schedules in AMI. In summary, our study aimed to determine the key genes that are involved in the regulation of AMI development by bioinformatics analysis, which could be served as potential targets for the diagnosis and treatment of AMI.

## Materials and methods

### Microarray data and analysis of differentially-expressed genes

The microarray expression data of peripheral blood samples was obtained from GEO (http://www.ncbi.nlm.nih.gov/geo) and GEO Series accession number GSE48060. This microarray profiling was acquired from human peripheral blood samples of 31 AMI patients and 21 controls. 52 samples were profiled using the chip-based platform GPL570 [HG-U133_Plus_2] Affymetrix Human Genome U133 Plus 2.0 Array (Suresh et al., 2014).

The use of gene expression data from whole blood instead of heart tissue samples may limit the ability of our approach to detect signatures in the specific diseased tissue. However, peripheral blood is more easily accessed than heart tissue and may provide specific insights into the immune and inflammatory response of AMI exacerbations.

Microarray data analysis was performed using R software and Bioconductor 3.3 (http://www.bioconductor.org/). By quantile normalization, base 2 logarithm conversions, background correction and normalization were performed on original expression data by Robust Multiarray Averaging (RMA) (Gautier et al., 2004); as a result 54,675 mRNAs were carried over for further analysis (Supplement Table 1).

We screened the significant DEGs from 31 AMI patients and 21 controls by Limma (linear models for microarray data) package of R 3.1.3 software (Sanges et al., 2007). The Fold change > 1.2 and *P* <0.05 was regarded as a threshold.

Cluster and TreeView are software programs for analyzing and visualizing DNA microarray data or other genomic data sets. In this study, Cluster analysis was used to differentially-expressed genes data and then visualize the data through TreeView.

### Gene ontology (GO) and pathway enrichment analyses

GO analysis was utilized to explain the primary function of the DEGs according to the GO database, which is the crucial functional classification of NCBI (Ashburner et al., 2000; Gene Ontology, 2006). Fisher's exact test was used to calculate the significance level (*P*-value) of each GO term to screen out the significance GO term of the DEGs enrichment. While *P* <0.05 was considered to be significant.

Analogously, pathway analysis was performed to discover the significant pathway terms of the DEGs according to the Kyoto Encyclopedia of Genes and Genomes (KEGG) (Kanehisa and Goto, 2000). We used the Fisher's exact test to identify the significant pathway terms; a *P* <0.05 was considered significant (Kanehisa et al., 2004; Draghici et al., 2007).

### Construction of gene function regulatory network (GO-tree)

The GO hierarchy was a directed acyclic graph (DAG), in which each term had a defined link with one or more other terms. A GO-Tree was constructed based on the GO DAG to supply clear data navigation and visualization. *P* <0.01 were selected for statistical significance of GO terms in GO analysis, and the GO-Tree was constructed using the up- and down-regulated DEGs in order to outline the functions that influenced AMI (Zhang et al., 2004).

Experimental genes also participated in many significant GO analyses. We organized the mutual regulation and affiliations between all GO analyses into the database based on a hierarchy of GO. By building a functional relationship network, it was possible to summarize the impact of the experimental function groups, as well as internal affiliation significant features. We considered the biological process (BP) terms of GO analysis to build a network of functional regulation (*P* <0.01).

### Construction of pathway action network (pathway-act-network)

The KEGG database included signal transduction, metabolism, cell cycle, membrane transport pathways and information of their interactions. The genes we selected may be related to two or more signaling pathways. Due to the same genes in different pathways, overlapping between pathways was inevitable. We selected the genes in enriched biological pathways and used Cytoscape for graphical representations of pathways (Shannon et al., 2003). By constructing the pathway action network, the signal transfer relations between the pathways was explored at a macro level. In order to look for the core pathways and regulatory mechanisms affected by the disease from multiple significant pathway terms, *P* <0.05 of pathway terms in pathway analysis were selected to construct a pathway action network.

### Co-expression network analysis

Gene co-expression network analyses (Kim et al., 2001) were carried out to reveal the interrelation between the DEGs, based on their normalized signal intensity in “AMI” and “Control” profiles. The Pearson correlation was calculated for each pair of genes, and the significantly correlated pairs were used to construct the co-expression network (Prieto et al., 2008). To trace the core regulatory genes in the networks, k-core scores were applied to simplify the analysis of graph topology. The k-core of a given gene indicated its core or nodal status linking other genes in the network to “k” (Ravasz et al., 2002; Barabási and Oltvai, 2004). Consequently, we applied k-core scores to identify genes with the highest networking degrees as key regulatory genes.

### Weighted gene co-expression network analysis

WGCNA is a common algorithm for constructing a co-expression network. We utilized the similarity of gene co-expression to define a network. When using *S*_*mn*_ to represent the gene co-expression similarity between genes *m* and *n*, we can then apply the power adjacency function for correlating adjacency of genes: *S*_*mn*_ = ${\left|\left(1+cor\left(x_{m},x_{n}\right)\right)/2\right|}^{\beta }$. In data processing, the genome-wide gene expression data was preliminarily filtered, followed by measuring the consistency of gene expression profiles by Pearson correlation, and finally using the power adjacent function to Pearson correlation matrix, data was transformed into weighted gene co-expression networks.

Intramodular connectivity (IC) describes the correlation degree of a gene with other genes in a given module, which can be understood as a measure of module membership (MM). Network module (Module) is a cluster of closely interconnected genes. During module detection, the adjacency matrix (which is a measure of topology similarity) is transformed into the topological overlap matrix (TOM), and modules are detected by cluster analysis (Zhao et al., 2014). We then analyzed the importance of genes by the *t*-test to determine whether the modules were associated with myocardial inflammation. Module eigengene (ME) refers to the first principal component gene of module expression matrix. It is considered the most representative of the module genes, which has important biological significance.

### Functional enrichment analysis of the network module genes

The online tool PANTHER was used to analyze the functional enrichment of network module genes that are associated with myocardial inflammation (*P* <0.05) (Mi et al., 2013).

### Identification of hub genes

Hub genes are described as the genes most closely associated with disease; they are connected to the highest degree of a series of genes in a module. To a certain extent, they are used to determine the character of modules, the hub genes of modules often have more biological significance compared with the hub genes of global networks (Jeong et al., 2001; Goh et al., 2007; Liang and Li, 2007). A gene is considered to be a hub gene if it has a unique character, such as high GS, high MM and high IC in the network (Zhang and Horvath, 2005). Gene significance (GS) showed the different IC of a gene in various networks, and MM described the significance of genes in the network. The IC of a gene suggested the connectivity with the other genes within network. We were therefore also able to identify the hub genes in modules through the GS, MM, and IC.

### Transcription regulatory network

JASPAR (http://jaspar.genereg.net) is an open-access database storing curated, non-redundant transcription factor (TF) binding profiles representing transcription factor binding preferences as position frequency matrices for multiple species in six taxonomic groups (Mathelier et al., 2016). Cytoscape is a visualization data integration package for biological networks based on the Java language (Smoot et al., 2011). We imported DEGs into the JASPAR database to obtain the interaction between transcription factors and their target genes (TG). Then, we used Cytoscape software to visualize these relationships and finally obtained the transcription factor-target gene regulatory network.

### Patients

The research object of this study with a total of 20, all are hospitalized patients with AMI in the department of cardiology of first hospital of Jilin university, and each was invited to join the study patients are required to sign a consent form. Our research protocol was approved by the ethical committee of the First Hospital of Jilin University. Among the 20 patients, 12 males, and 8 females, aged 57 ± 9 years. Inclusion criteria: chest pain or distress within 24 h duration > 30 min, and myocardial enzymes CK-MB and cTNT higher than the normal range online. While excluding myocarditis and other diseases caused by chest pain or distress; exclude the patients with history of renal failure; patients with advanced liver disease; patients with malignant tumors and patients with other inflammatory diseases, such as psoriasis, rheumatoid arthritis and so on.

### Quantitative real-time RT-qPCR

According to the microarray results, the 8 most dysregulated mRNAs were chosen for further validation by RT-qPCR in AMI patients vs. healthy controls. Blood samples were collected and PBMCs were isolated. Total RNAs were isolated from PBMCs using Trizol reagent (Invitrogen), and complementary DNA was synthesized using the *TransScript*®Frist-Strand cDNA Synthesis SuperMix(Transgen, China)according to the manufacturer's instructions. Primer sets for selected genes were designed by Sangon Biotech (Shanghai, China); their sequences and reaction conditions are available in Table 1. Each sample was run in triplicate in 96-well plates using LightCycler®96 and FastStart Essential DNA Green Master (Roche Diagnostics GmbH, Germany). Quantification cycles (Cq) were calculated using the fit point method (LightCycler®96 Software, Version 1.1 provided by Roche). The expression data were normalized to the reference glyceraldehyde-3-phosphate dehydrogenase (Gapdh). All experiments (sample collection, preparation and storage, primer design, qPCR normalization) were performed according to the MIQE guidelines.

**Table 1:** **PCR primers for quantitative real-time PCR**.

**Gene**	**Primer sequence (5′→3′)**	
GAPDH	F : CCACATCGCTCAGACACCAT	R : GGCAACAATATCCACTTTACCAGAGT
AQP9	F : GCCATCGGCCTCCTGATTAT	R : GCCCACTACAGGAATCCACC
DYSF	F : CCTGCCATGTTTCCCTCCAT	R : AGGTAGGTGGTAGCCACGAT
GZMA	F : CTGGAAGCCCTTTGTTGTGC	R : CACGAGGGTCTCCGCATTTA
NCF4	F : TCCCAGATGAGCCACAATGC	R : ATAGGGGAGTGCTGCTGAGA
NFIL3	F : CCGAGAACGTCGGAAACTGA	R : TTGGCTTTGATCCGGAGCTT
PRF1	F : ACCTTCATCCAAGCATGGGG	R : TATTGTCCCACACGGTGCTC
PTGDR	F : CCTTCTTTGGGCTCTCCTCG	R : GAACTTCCCGAAGCCCATGA
TBX21	F : CCACCTGTTGTGGTCCAAGT	R : GGGAACATCCGCCGTCC

## Results

### DEGs selection and hierarchical clustering analysis

We used R software and corrected batch effect by Bayesian methods, then removed the probes without corresponding annotation information. To determine the expression values of each gene, we used multiple GSE48060 probes corresponding to the median expression value of that gene, and finally obtained the expression profile of 54,675 genes (Supplement Table 1).

**Figure 1:**
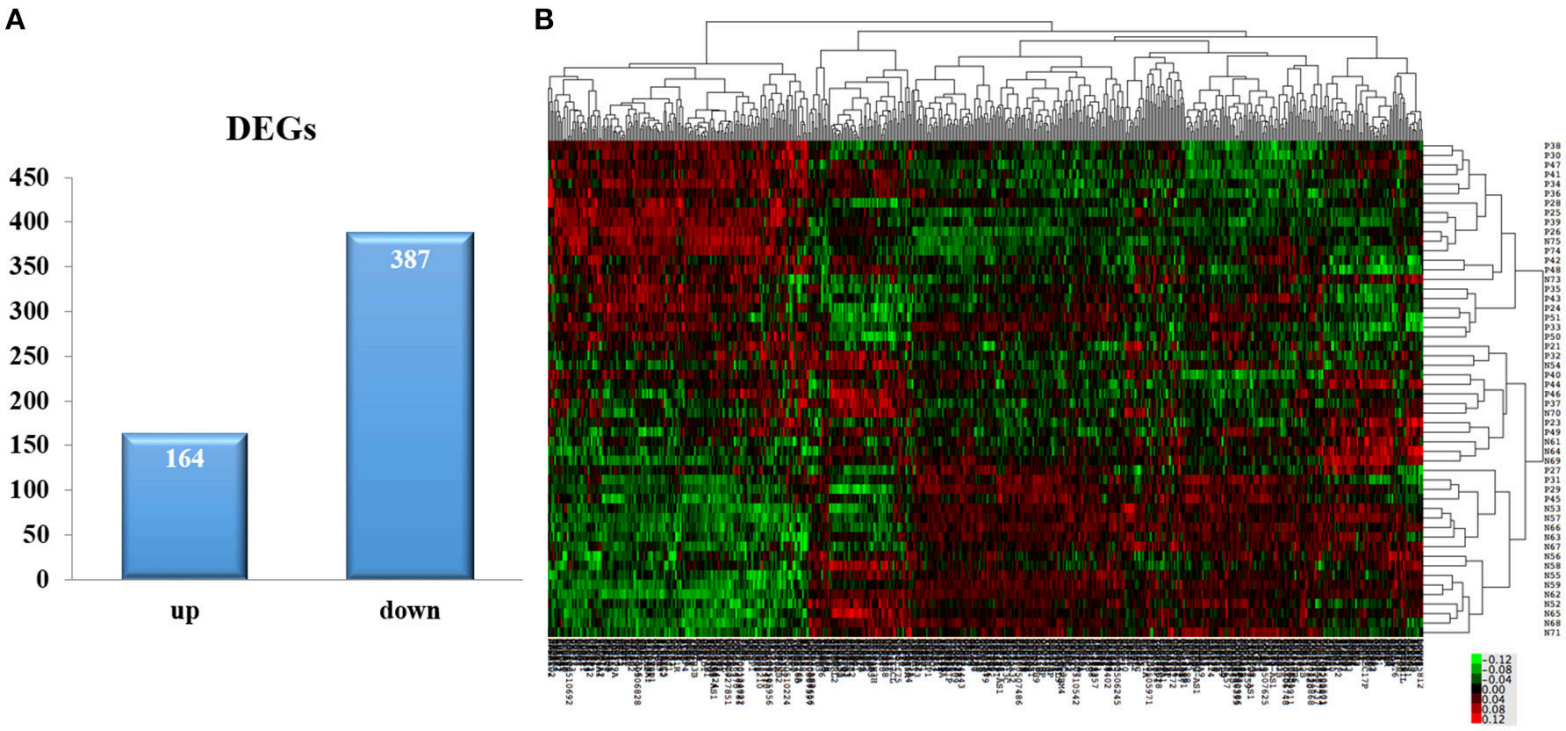
**DEGs selection and hierarchical clustering analysis. (A)** The Limma algorithm was applied to filter the DEGs; we selected the DEGs according to *P* <0.05 and Fold change > 1.2. **(B)** DEGs can be effectively divided into AMI and control groups. Red indicates that the gene that is upregulated and green represents down-regulated genes.

By using algorithms provided in the Limma package, we calculated the data and obtained with lists of differentially expressed genes. A total of 551 DEGs were identified between the AMI group and the control group, including 164 upregulated and 387 down-regulated genes (*P* <0.05, Fold change > 1.2, Figure 1A, Supplement Table 2). Hierarchical clustering analysis was obtained for the 551 DEGs from the 52 samples of the AMI and control groups. The general gene expression patterns were evidently different in the two groups by TreeView (Figure 1B).

### GO enrichment analysis of DEGs and GO-tree

**Figure 2:**
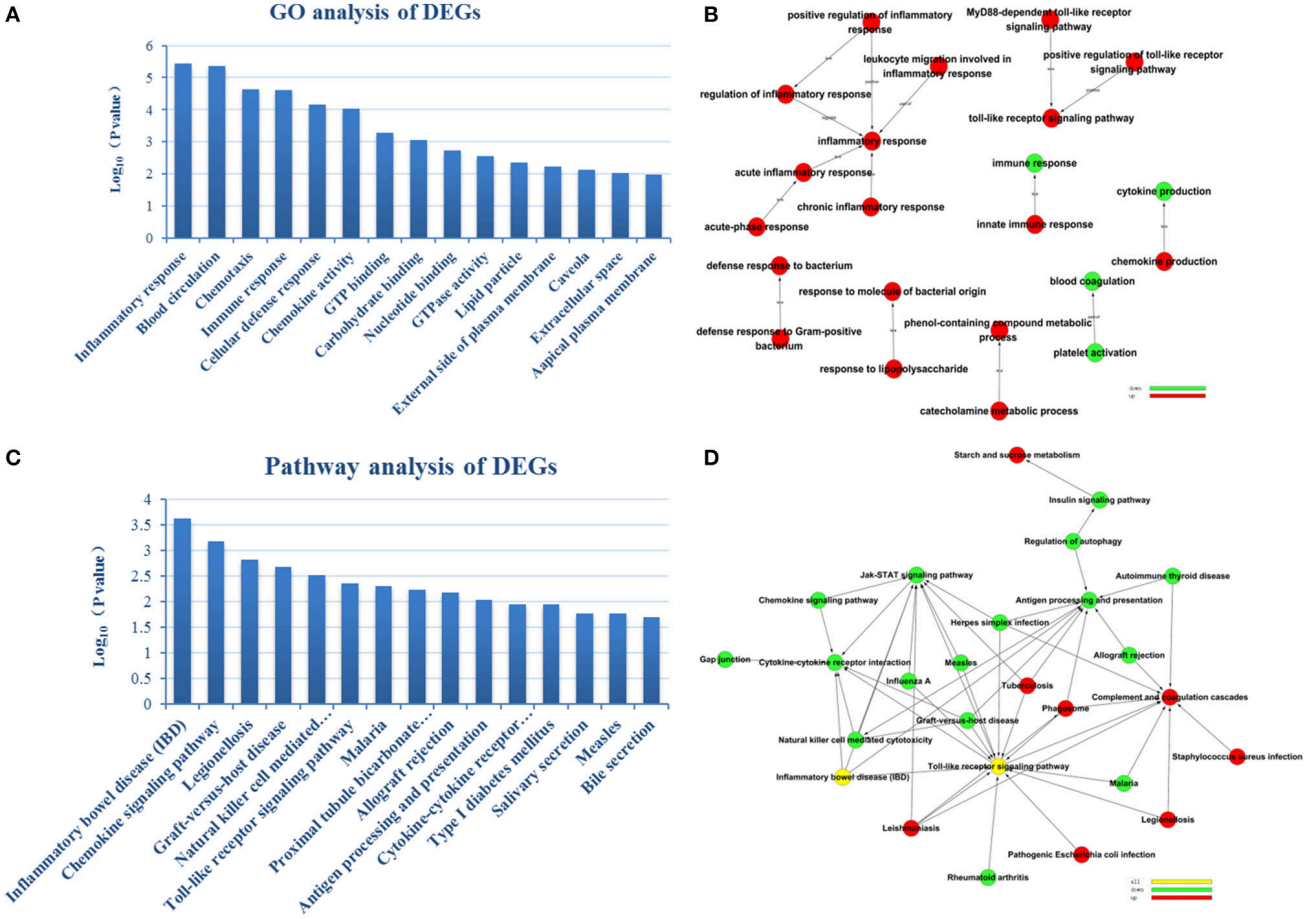
**GO enrichment analysis and GO-Tree. (A)** The significant GO terms that conformed to a *P* <0.05 were screened. **(B)** GO-Tree: red circles represent the upregulated genes involved in GO terms, and green circles represent the down-regulated genes involved in GO terms. **(C)** We used the Fisher's exact test to select the significant pathway, identified by a *P* <0.05. **(D)** Pathway-act-network. *P* <0.05. Red circles represent the upregulated genes involved in pathway terms, and green circles represent the down-regulated genes involved in pathway terms.

To reveal AMI–related biological processes, we conducted a functional enrichment analysis. GO enrichment analysis of 551 DEGs was performed. The most GO terms of biological processes were associated with inflammation, including the inflammatory response, immune response, cellular defense response and chronic inflammatory response in the AMI group (Figure 2A). In the cellular component category, enriched GO terms were mainly associated with lipid particles, the external side of the plasma membrane and the extracellular space. In the molecular function category, GO terms enriched for DEGs in AMI included chemokine activity, GTP binding and carbohydrate binding (Table 2). We constructed gene function regulatory networks (GO-Tree) for the significant GO terms (*P* <0.01) of the category of biological processes in GO analysis in order to explore the intrinsic link among gene function (Figure 2B). We then found the hierarchical tree relationships between gene function significantly (Supplement Table 3). The analysis showed that the gene function cascade eventually induced inflammation during AMI.

**Table 2:** **Gene ontology (GO) enrichment analysis (top 5 significantly enriched biology terms)**.

**ID**	**Category**	**Term**	**Count**	***p*-value**
BP (biological process)	GO:0006954	Inflammatory response	25	3.72505E-06
	GO:0008015	Blood circulation	9	4.228E-06
	GO:0006935	Chemotaxis	15	2.2594E-05
	GO:0006955	Immune response	27	2.44648E-05
	GO:0006968	Cellular defense response	9	6.92717E-05
MF (molecular function)	GO:0008009	Chemokine activity	8	9.08077E-05
	GO:0005525	GTP binding	23	0.000533266
	GO:0030246	Carbohydrate binding	13	0.000880498
	GO:0000166	Nucleotide binding	79	0.001891473
	GO:0003924	GTPase activity	15	0.002768396
CC (cellular component)	GO:0005811	Lipid particle	6	0.004507736
	GO:0009897	External side of plasma membrane	12	0.005867119
	GO:0005901	Caveola	6	0.007176272
	GO:0005615	Extracellular space	36	0.009088137
	GO:0016324	Apical plasma membrane	13	0.010146435

### Pathway enrichment analysis of DEGs and pathway-act-network construction

To screen the significant enrichment of DEGs in pathway terms, Fisher's exact test was used to calculate the significance level of the pathway (*P* <0.05). According to KEGG databases, we performed pathway annotation of DEGs and obtained DEGs involved in all pathway terms (Figure 2C, Table 3). The DEGs of AMI were enriched mainly in inflammatory bowel disease (IBD), the chemokine signaling pathway, natural killer cell mediated cytotoxicity, toll-like receptor signaling pathway and cytokine-cytokine receptor interactions. As there was cross-talk between the pathway terms, in order to search the transitive relation between signaling pathways, we constructed pathway action networks (pathway-act-network) for the significant pathway terms (*P* <0.05) (Figure 2D, Supplement Table 4). There were interactions in multiple pathways, demonstrated by the pathway-act-network, which ultimately impacted activation of multiple pathway terms including the JAK-STAT signaling pathway, toll-like receptor signaling pathways and antigen processing and presentation.

**Table 3:** **KEGG enrichment analysis of genes (top 15 significantly enriched pathway terms)**.

**ID**	**Category**	**Term**	**Count**	***p*-value**
KEGG_PATHWAY	PATH:05321	Inflammatory bowel disease (IBD)	9	0.000234194
	PATH:04062	Chemokine signaling pathway	15	0.000660309
	PATH:05134	Legionellosis	7	0.001477228
	PATH:05332	Graft-vs.-host disease	6	0.002078294
	PATH:04650	Natural killer cell mediated cytotoxicity	11	0.002955348
	PATH:04620	Toll-like receptor signaling pathway	9	0.004404204
	PATH:05144	Malaria	6	0.004876618
	PATH:04964	Proximal tubule bicarbonate reclamation	4	0.005750295
	PATH:05330	Allograft rejection	5	0.006633034
	PATH:04612	Antigen processing and presentation	7	0.009121472
	PATH:04060	Cytokine-cytokine receptor interaction	15	0.011203809
	PATH:04940	Type I diabetes mellitus	5	0.011308703
	PATH:04970	Salivary secretion	7	0.016840879
	PATH:05162	Measles	9	0.016897245
	PATH:04976	Bile secretion	6	0.019514032

### Gene co-expression network

**Figure 3:**
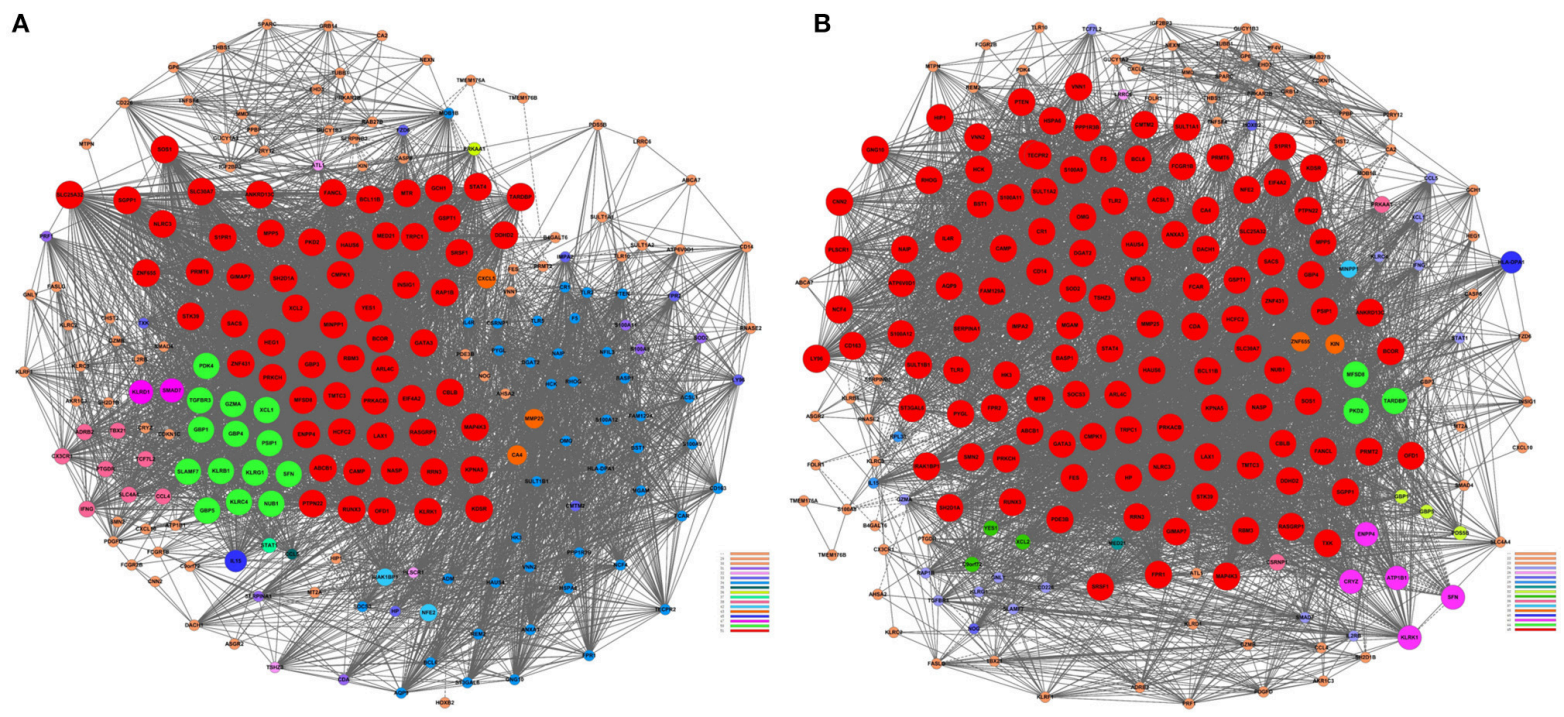
**Gene co-expression network analyses**. The Pearson correlation was calculated for each pair of genes, and the significantly correlated pairs were used to construct the co-expression network (*P* <0.05). **(A)** AMI. **(B)** Control. Red nodes represent the key regulatory genes with the highest K-Core. The node color represents the K-Core. The node size represents the K-Core power, and the edges between two nodes represent the interactions between the genes.

We performed a gene co-expression network analysis to investigate the phenotypic change of genes associated with AMI (Kumari et al., 2012; Villa-Vialaneix et al., 2013). Twelve genes from the AMI and control groups were chosen as key regulatory genes (|*Dif*_*Kcore*| > 30), and it was shown that there was a significant change in the expression pattern of genes in the AMI group (Supplement Table 5), namely *CNN2* (calponin 2), *CRYZ* (quinone oxidoreductase), *SULT1A1* (sulfotransferase 1A1), *SULT1A2* (sulfotransferase 1A2), *PRMT2* (protein arginine methyltransferase 2), *ATP1B1* (ATPase, Na^+^/K^+^ transporting, beta 1 polypeptide), *CX3CR1* (CX3C chemokine receptor 1), *GCH1* (GTP cyclohydrolase 1), *INSIG1* (insulin-induced gene protein), *CXCL5* (C-X-C motif chemokine 5), *GBP3* (guanylate-binding protein 3) and *HEG1* (heart development protein with EGF-like domains 1).We hypothesized that the 12 key regulatory genes are likely to be closely related to the occurrence and development of AMI (Figure 3).

However, traditional studies of gene co-expression patterns mainly utilize the Pearson coefficient to describe the correlation between genes, and tend to determine the presence of a co-expression network using the Pearson coefficient and FDR threshold (also known as hard threshold) (Butte and Kohane, 2000; Carter et al., 2004; Prieto et al., 2008). The two genes are considered to be connected when the correlation coefficient of two genes is equal to or greater than this threshold (for example, 0.8). However, there is a significant limitation to this method, i.e., we have no evidence to identify whether the coefficient 0.8 or 0.79 of two genes has a significant difference. The above algorithm cannot avoid this situation. WGCNA is different from the traditional gene-gene correlation coefficient matrix, and introduces a soft-threshold method that avoids this limitation. In WGCNA, the correlation coefficient in genes of a gene co-expression matrix is obtained by weight calculation. The standard of weighting is the connections between genes, which should conform to the distribution of scale-free networks, obeying a power-law distribution. In short, the correlation coefficient of each gene pair was the β-th power of the exponent operation, which was the weight calculation and β was known as the soft threshold.

### Weighted gene co-expression networks

We selected a suitable weighted parameter of adjacency function, which is the soft-threshold β, before constructing the weighted co-expression network. After the calculation, we selected the correlation coefficient close to 0.8, soft-threshold β = 9 to construct gene modules using the WGCNA package.

**Figure 4:**
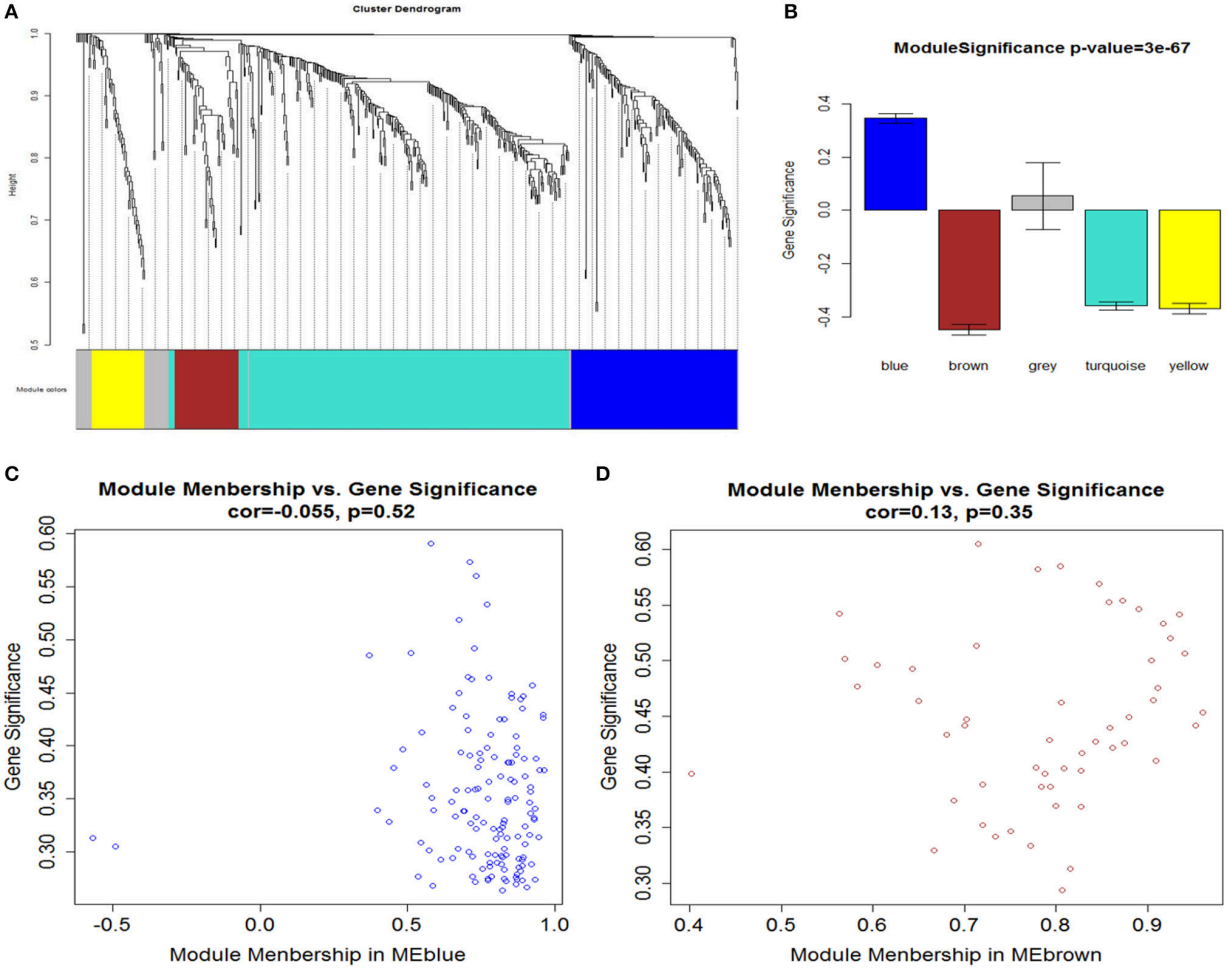
**(A)** Network analysis of gene expression in AMI identifies distinct modules of co-expression genes. **(B)** Correlation between the gene modules and immune and inflammatory responses. Scatter plot of MM vs. GS in **(C)** blue and **(D)** brown modules. Cor represents an absolute correlation coefficient of GS and MM; *P*-value for significance assessment. It follows that in both modules, GS and MM have a high correlation. In the high correlation of image: upper right node (gene) and immune and inflammatory responses, on the other hand in the module also has an important significance.

After determining the soft threshold, a total of 551 DEGs were used to construct weighted gene co-expression networks. According to the basic idea of WGCNA, we calculated the correlation matrix and adjacency matrix of the gene expression profile of the AMI and control groups, and then transformed them into a topological overlap matrix (TOM), and obtained a system clustering tree of DEGs on the basis of gene-gene non-similarity ($d_{ij}^{\omega }$ = 1− ω_*ij*_). Together with the TOM, we performed the hierarchical average linkage clustering method to identify the gene modules of each gene network (deep split = 2, cut height = 0.99). In both the AMI and control groups, a total of five gene modules were recognized by the dynamic tree cut (Figure 4A). Genes that not belong to any modules were housed in the gray module. The gray gene modules were ignored in this study.

### GO enrichment analysis of the gene modules

For a preliminarily test to assess whether the network was acceptable, we derived the corresponding genes of each module, and PANTHER was used for the functional enrichment analysis of GO terms, *P* <0.05 (Mi et al., 2013). For the blue module, genes involved in the inflammatory response (GO: 0006954, *P* = 1.81E-07), immune response (GO: 0006955, *P* = 1.54E-05), defense response (GO: 0006952, *P* = 5.12E-05) and immune system process (GO: 0002376, *P* = 1.35E-03) were significantly enhanced (Table 4). It is suggested that this module is closely related to the occurrence and development of the immune and inflammation responses in AMI. Meanwhile, by analyzing the brown module, it is evident that the module gene is mainly concentrated in the immune response (GO: 0006955, *P* = 8.83E-06), regulation of lymphocyte chemotaxis (GO: 1901623, *P* = 1.05E-05), defense response (GO: 0006952, *P* = 2.03E-05), response to stimulus (GO: 0050896, *P* = 9.99E-05) and immune system process (GO: 0002376, *P* = 1.53E-04) (Table 4). These results indicate that the module may be closely related to the immune response during AMI. The gene modules that we obtained have biological significance, so we named the blue and brown modules as immune modules. The functional enrichment analysis of blue and brown modules indicates that the immune response and inflammation would immediately follow the onset of AMI, which is consistent with previous reports (Fang et al., 2015).

**Table 4:** **Gene ontology (GO) enrichment analysis in gene modules (top 8 significantly enriched biology terms)**.

**Module**	**ID**	**Category**	**Term**	**Count**	***p*-value**
Blue	BP (biological process)	GO:0006954	Inflammatory response	19	1.81E-07
		GO:0006955	Immune response	30	1.54E-05
		GO:0006952	Defense response	30	5.12E-05
		GO:0002376	Immune system process	34	1.35E-03
		GO:0050896	Response to stimulus	74	4.54E-03
		GO:0042742	Defense response to bacterium	10	5.12E-03
		GO:0006950	Response to stress	44	2.17E-02
		GO:0098542	Defense response to other organism	12	2.58E-02
Brown	BP (biological process)	GO:0006955	Immune response	45	1.22E-11
		GO:0006952	Defense response	45	8.05E-11
		GO:0002376	Immune system process	51	8.13E-09
		GO:0006954	Inflammatory response	23	1.02E-08
		GO:0050896	Response to stimulus	105	7.10E-08
		GO:0006950	Response to stress	64	1.77E-06
		GO:0009605	Response to external stimulus	45	4.41E-06
		GO:0033993	Response to lipid	26	6.67E-06

A major goal of our research was to analyze the degree of correlation between genes and disease, and to ascertain the significance of genes in the corresponding modules. We defined GS as the association of a gene with immune and inflammatory responses. Then we calculated the mean GS of all genes in the module, which is module significance (MS), and described subsequently the relationship between modules and the inflammatory response (Langfelder and Horvath, 2008). Figure 4B showed the correlation between the various modules and immune/inflammatory responses, which shows that the most relevant modules to the immune and inflammatory responses were the blue and brown modules. For a particular gene, to evaluate its involvement in the given module, we analyzed the correlation between its expression profile and ME profile in all samples. We calculated the MM of all the genes in the corresponding module to learn their significance within the given modules. We identified modules (blue and brown) that showed a high correlation with immune and inflammatory reactions, and GS and MM of each gene are shown in Figures 4C,D.

### Hub genes associated with the occurrence of AMI

**Figure 5:**
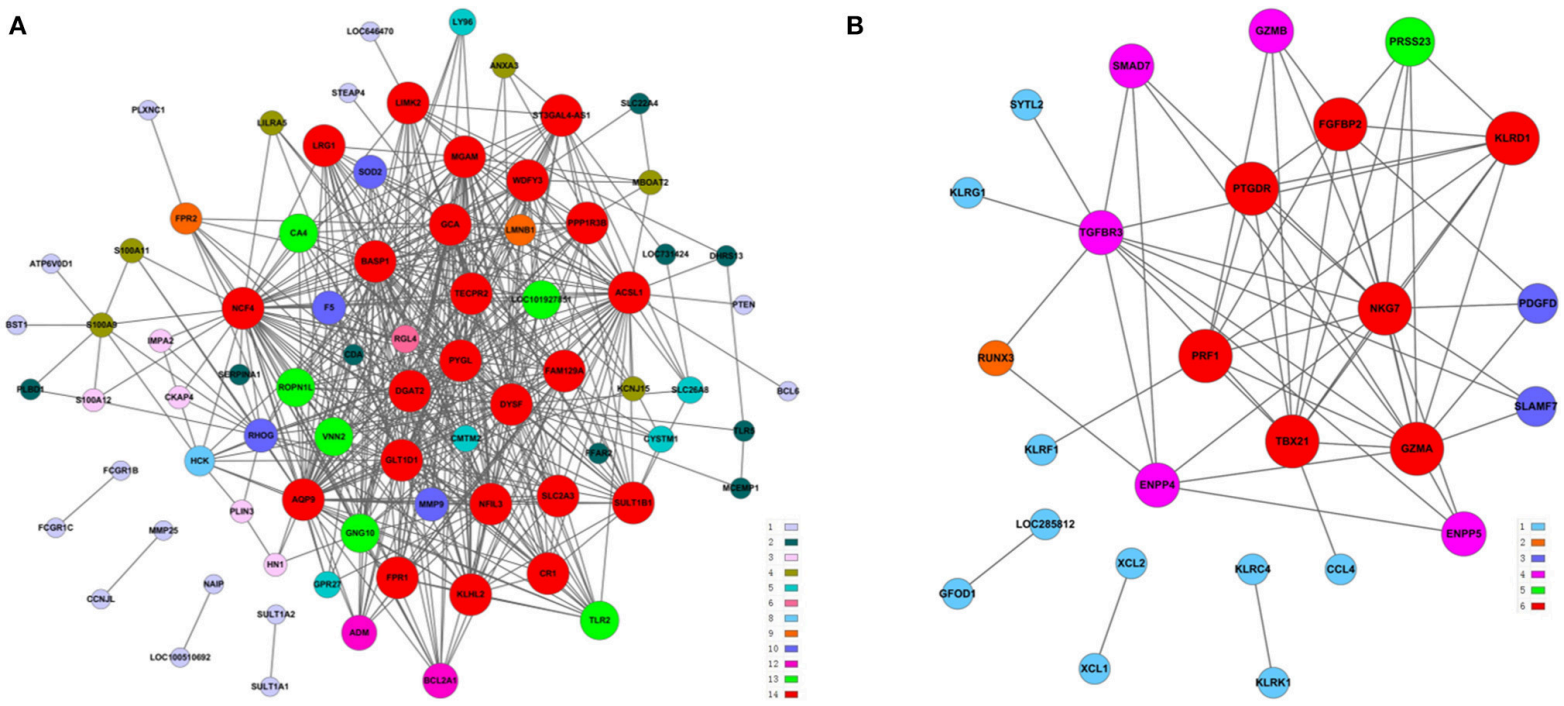
**Construction of a co-expression network of module genes. (A)** Blue module; **(B)** brown module. Red nodes represent the hub genes with the highest K-Core and MM. The node color represents the K-Core. The node size represents the K-Core power, and the edges between two nodes represent the interactions between the genes.

The above results suggested that the occurrence and development of AMI were closely related to the immune and inflammatory responses. To screen genes that were most relevant to immune and inflammatory responses, we constructed the gene co-expression network of immune module genes. There was a positive correlation between MM and IC in the modules, but not GS. Therefore, we selected the hub genes according to the IC and MM values of each gene module (Table 5). Eight hub genes were selected, including *NCF4, AQP9, NFIL3, DYSF, GZMA, TBX21, PRF1*, and *PTGDR*. Combined with previous reports, we hypothesized that these hub genes were closely relevant to immune and inflammatory responses when AMI occurs. The co-expression network of module genes was then constructed to obtain the degree of connection between genes in the module (Figures 5A,B).

**Table 5:** **The hub genes in the brown and blue module (MM > 0.9)**.

**Module**	**Gene symbol**	**Description**	**Log2FC**	***P*-value**	**FDR**	**Style**	**Intramodular connectivity**	**MM module**	**K-core**
Brown	NKG7	Protein NKG7	−0.59977622	4.57E-04	0.081114	Down	12.92371	0.9597275	6
Brown	GZMA	Granzyme A	−0.5650966	6.70E-04	0.089693	Down	12.57077	0.9525589	6
Brown	TBX21	T-box 21 variant	−0.548076	7.24E-05	0.035039	Down	11.24149	0.9409293	6
Brown	PRF1	Perforin-1	−0.56140565	1.81E-05	0.017504	Down	10.67641	0.934967	6
Brown	KLRD1	Natural killer cells antigen CD94	−0.76968129	4.09E-05	0.027231	Down	10.22693	0.9252181	6
Brown	PTGDR	Prostaglandin D2 receptor	−0.59329129	2.47E-05	0.021076	Down	9.142072	0.916501	6
Blue	NCF4	Neutrophil cytosol factor 4	0.451293433	0.0043104	0.177619	Up	33.50208	0.9637546	14
Blue	DYSF	cDNA FLJ55344, highly similar to Dysferlin	0.460010493	0.0010058	0.103739	Up	32.4434	0.9613569	14
Blue	GLT1D1	cDNA FLJ51476	0.44564518	0.0011065	0.105197	Up	31.61535	0.9595483	14
Blue	AQP9	cDNA FLJ50860, highly similar to Aquaporin-9	0.531897672	0.0042735	0.176717	Up	32.72345	0.9488406	14
Blue	PYGL	Alpha-1,4 glucan phosphorylase	0.399190191	0.0187515	0.272497	Up	30.81584	0.9454835	14
Blue	DGAT2	Diacylglycerol O-acyltransferase 2	0.422619622	0.0032528	0.163039	Up	29.01008	0.9349146	14
Blue	BASP1	Brain acid soluble protein 1	0.475512299	0.0103385	0.226315	Up	30.82379	0.9330127	14
Blue	GCA	cDNA FLJ52146, highly similar to Grancalcin	0.281390861	0.0423062	0.332922	Up	30.09932	0.9316431	14
Blue	NFIL3	Nuclear factor, interleukin 3 regulated, isoform CRA_a	0.477300573	0.0130679	0.244778	Up	28.80993	0.9294694	14
Blue	ACSL1	cDNA FLJ76467, highly similar to Homo sapiens acyl-CoA synthetase long-chain family member 1 (ACSL1), mRNA	0.518836972	0.0125705	0.242483	Up	30.37111	0.9289425	14
Blue	SULT1B1	Sulfotransferase family cytosolic 1B member 1	0.519448931	4.21E-04	0.077407	Up	26.96646	0.9233878	14
Blue	KLHL2	Kelch-like protein 2	0.343649623	0.0317126	0.30949	Up	26.99282	0.9210025	14
Blue	MGAM	Maltase-glucoamylase (Alpha-glucosidase), isoform CRA_a	0.507642227	0.0313529	0.308545	Up	28.52488	0.9204113	14
Blue	SLC2A3	cDNA FLJ57557, highly similar to Solute carrier family 2, facilitated glucose transporter member 3	0.274927717	0.0484124	0.347472	Up	24.5968	0.9038037	14

### Transcription regulatory network

**Figure 6:**
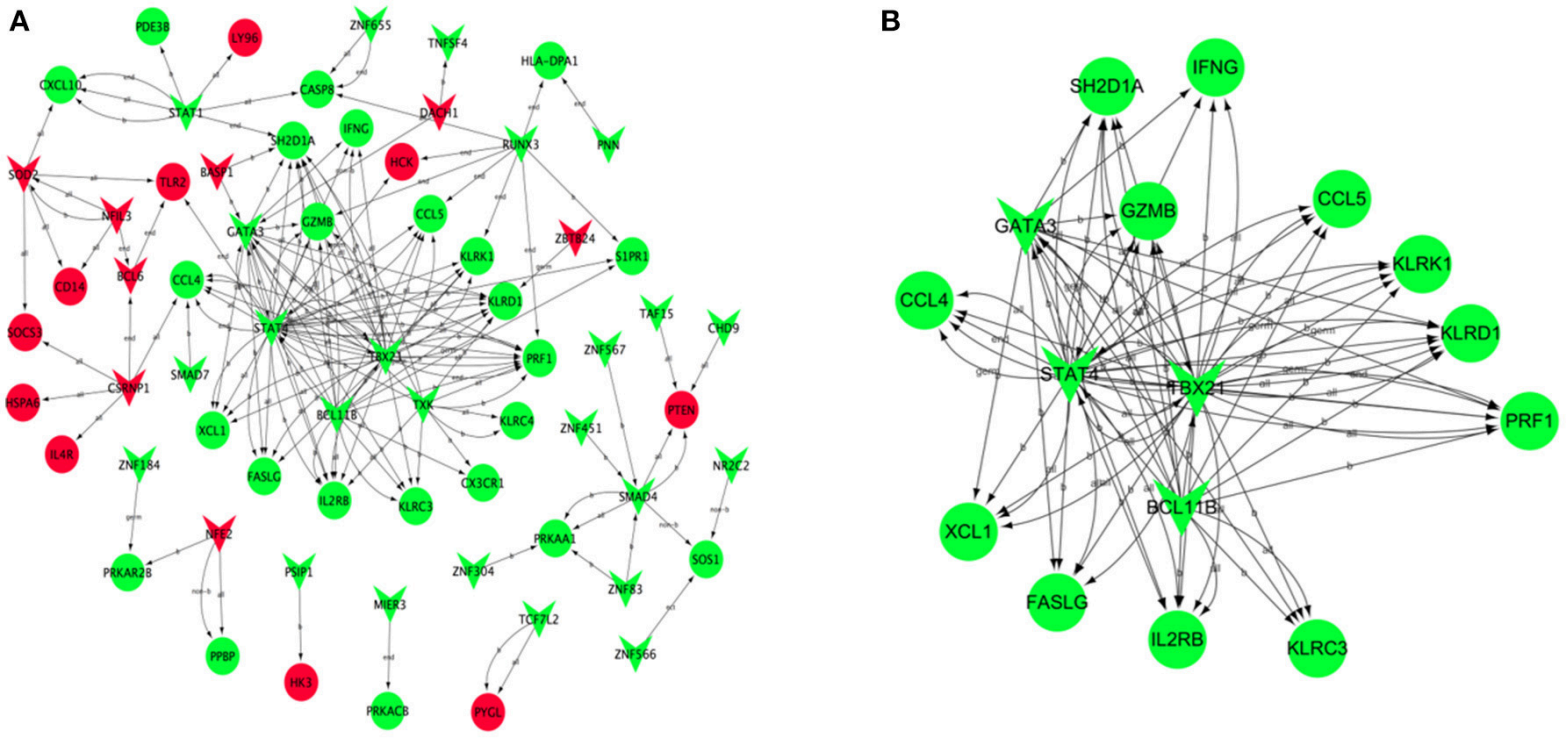
**Transcription regulatory network of DEGs**. Based on JASPAR database, the transcription regulatory network constructed by DEGs included 66 nodes **(A)**. **(B)** Construction of transcription regulatory network centered around TBX21. Red V-type represents up-regulated TF, green V-type represents down-regulated TF; red circles represent up-regulated TG, green circles represent the down-regulated TG.

Based on JASPAR database, the transcription regulatory network of DEGs was constructed and included 66 nodes (Figure 6A). There were 30 transcription factors, and the core transcription factors were SATA4, BCL11B, GATA3, and TBX21, which have the regulatory relationship with the most target genes. (TG > 10, Supplement Table 6). Combined with the results of WGCNA analysis, we found that the transcription factor TBX21 was a hub gene. We screened all target genes associated with the transcription factor TBX21 and constructed a transcriptional regulatory network centered around TBX21 (Figure 6B). Significantly, TBX21 was directly related to the transcription factors SATA4, BCL11B, and GATA3.Interestingly, these four transcription factors were also directly related to PRF1, and PRF1 was also a hub gene.

### Validation of microarrays with RT-qPCR

**Figure 7:**
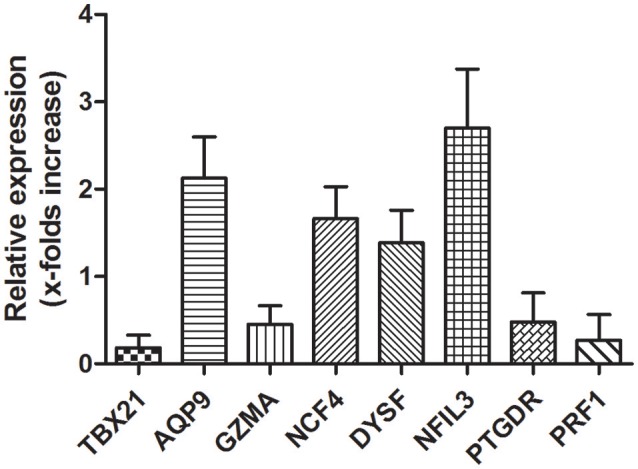
**Validation of microarray data with RT-qPCR**. Several hub genes identified in microarray data are dysregulation in AMI patients. mRNA expression of hub genes identified in microarray data validated by RT-qPCR is shown. Total RNAs were isolated from PBMCs or AMI patients and healthy donors. Reverse-transcribed to cDNA and used as template for RT-qPCR analysis. Relative Expression of each gene in PBMCs from healthy donors were considered as 1.

RT-qPCR was used to validate the microarray data. To verify the main conclusion drawn from the microarray results for peripheral blood samples, the expression levels of genes coding for *NCF4, AQP9, NFIL3, DYSF, GZMA, TBX21, PRF1*, and *PTGDR* were determined. Overall, the RT-qPCR results were qualitatively consistent with the results of the microarray analysis. However, the RT-qPCR analysis tended to give higher up-regulation levels than those calculated from the microarray data. The highest change was found for *TBX21, PRF1*, and *NFIL3* (Figure 7).

## Discussion

Although the appropriate thromboprophylaxis treatments for AMI have reduced successfully the incidence and mortality of AMI to some extent (Lau and Lip, 2014), effective methods for preventing and diagnosing AMI are still controversial. In our study, we use a systematic biology approach to identify 551 DEGs in blood samples from AMI patients compared with control groups. For enrichment analysis of pathways, DEGs were mainly involved in IBD, the chemokine signaling pathway, toll-like receptor signaling pathway and cytokine-cytokine receptor interactions. From the functional enrichment and GO-Tree analysis, we found that the major biological processes are the inflammatory response, chemotaxis, the immune response and the cellular defense response when AMI occurs; these are functional annotations associated with the immune and inflammatory responses.

In the WGCNA, we identified five gene modules based on 551 DEGs. Each module contained 44–279 genes; only 36 genes failed to be assigned to any of the gene modules. By functional enrichment analysis, the modules have obvious biological significance, the genes of blue and brown modules were significantly enriched in immunity and inflammation-related biological processes. The majority of genes within the turquoise module were involved in mRNA processing and transcription, and the yellow module genes were involved mainly in platelet coagulation and activation.

Through the enrichment function analysis of the module genes, we determine that accompanied by the activation of inflammatory response signal pathways and the initiation of immune system when AMI occurs. After the analysis of WGCNA, we selected the blue and brown modules, which were closely related to inflammatory reaction, as the main research objects. Eight potential hub genes related to AMI were screened from blue and brown modules, namely *NCF4, AQP9, NFIL3, DYSF, GZMA, TBX21, PRF1*, and *PTGDR*. Subsequently, RT-qPCR was used to verify the expression of the eight hub genes in peripheral blood of AMI patients. Our results suggested that the expression was consistent with the bioinformatics analysis results. Wherein the most obvious change of hub genes was *TBX21* and *PRF1*, which expression were significantly down-regulated in peripheral blood of AMI patients. In combination with the results of transcriptional regulatory network analysis, we hypothesized that transcription factor TBX21 and target gene PRF1 may be the diagnostic biomarkers of AMI and merit further explored.

Gene *TBX21* can encode a transcription factor named T-bet, whose main function is to suppress GATA-3 expression and prevent Th1 to Th2 cell differentiation (Szabo et al., 2000). It is well known that patients with AMI can develop subsequently cardiac arrest (Vanbrabant et al., 2006). Upon cardiac arrest, an abnormal stress response occurs and causes the change of transcription factor T-bet/GATA-3, resulting in the imbalance of Th1/Th2. The Th2-type cytokine-induced waterfall-like cascade is then initiated, which results in multiple organ failure, eventually leading to death. This has also been confirmed by a porcine model of cardiac arrest that lowers GATA-3 and T-bet levels, which alters the drifting Th1/Th2 cells and causes the immune imbalance of myocardial tissue after cardiopulmonary resuscitation (CPR) (Gu et al., 2013).

Perforin (PRF1) can be secreted by NK cells, CTL cells, γδ^+^T and regulatory T cells. PRF1 is a protein in the membrane attack complex/PR (FMACPF) superfamily (Lichtenheld et al., 1988; Shinkai et al., 1988), which is a highly conserved glycoprotein. Due to its important functions in immune surveillance and immune regulation, the malfunction of PRF1 has been found to be involved in many diseases (Baran et al., 2009). Thiery and Lieberman showed that PRF1 can activate clathrin- and dynein-dependent endocytosis, and inhibition of this endocytosis pathway may trigger apoptotic cell death (Thiery and Lieberman, 2014). In a study of 495 patients with cardiomyopathy, the infiltration of perforin-positive cells in the myocardium could serve as a predictor for long-term prognosis of patients; the presence of perforin-positive infiltration in myocardial cells indicates an adverse left ventricular ejection fraction (LVEF) course (Escher et al., 2014). We speculate that the abnormal expression of perforin may be a major cause of progressing LV dysfunction in AMI.

There is no report about the relationship between the TBX21 and PRF1 by far. It may be the focus of follow-up work that the potential regulatory role of TBX21 on PRF1 or other target genes in AMI. The highlight of this study was to identify several hub genes associated with AMI through the network analysis and to validate the expression levels of these hub genes by RT-qPCR experiments, including TBX21 and PRF1. We hypothesized that TBX21 and PRF1may be potential candidates for diagnostic and possible regulatory targets in the diagnosis and treatment of AMI. Our results provide us a direction for subsequent research in exploring thoroughly the gene regulatory mechanisms of AMI and its targeted therapy.

## Ethics statement

This study was carried out in accordance with the recommendations of ethics committee of First Hospital of Jilin University with written informed consent from all subjects (2015-019-12). All subjects gave written informed consent in accordance with the Declaration of Helsinki.

## Author contributions

JC participated in the design and execution of the study, conducted data analysis and interpretation, and drafted the manuscript. LY, SZ, and XC contributed to the design, execution and data interpretation. All authors participated in modifying and editing the manuscript, and the final manuscript has been read and approved by all authors.

### Conflict of interest statement

The authors declare that the research was conducted in the absence of any commercial or financial relationships that could be construed as a potential conflict of interest.
