# Supplementary material for: Network Analysis-Based Approach for Exploring the Potential Diagnostic Biomarkers of Acute Myocardial Infarction
Source: Front Physiol. 2016 Dec 9;7:615. doi: 10.3389/fphys.2016.00615 (PMC5145872; doi:10.3389/fphys.2016.00615)
Supplement: Supplementary file 2 [file Table2.PDF]

Table 2 Differentially-expressed genes in the peripheral blood of patients with AMI (P-Value<0.05 , Fold change＞1.2)

AccID	KeggID	Description	Fold change	P-Value	Style
**KLRB1**	hsa:3820	cDNA FLJ75274, highly similar to Homo sapiens killer cell lectin-like receptor subfamily B, member 1(KLRB1), mRNA	1.708684026	8.55E-07	down
**INSIG1**	hsa:3638	Insulin-induced gene protein	1.340829077	1.15E-06	down
**RABGAP1L**	hsa:9910	Rab GTPase-activating protein 1-like	1.370194864	2.33E-06	down
**FASLG**	hsa:356	Tumor necrosis factor ligand superfamily member 6	1.353620124	2.89E-06	down
**MMD**	hsa:23531	cDNA FLJ51447, highly similar to Monocyte to macrophage differentiation protein	1.50571908	4.12E-06	down
**KLRF1**	hsa:51348	Killer cell lectin-like receptor subfamily F member 1	1.769107949	4.82E-06	down
**SEC14L1P1**	hsa:729799	SEC14-like 1 pseudogene 1	1.216873846	4.92E-06	down
**HEG1**	hsa:57493	Protein HEG homolog 1	1.215641907	4.93E-06	down
**GOLGA8H**	hsa:728498	Golgin subfamily A member 8H	1.360585865	4.97E-06	down
**GART**	hsa:2618	cDNA FLJ51866, highly similar to Trifunctional purine biosynthetic protein adenosine-3	1.321976854	7.72E-06	down
**GZMB**	hsa:3002	Granzyme B (Granzyme 2, cytotoxic T-lymphocyte-associated serine esterase 1), isoform CRA_b	1.64901253	1.01E-05	down
**IL2RB**	hsa:3560	Interleukin-2 receptor subunit beta	1.417057745	1.15E-05	down
**SMAD7**	hsa:4092	cDNA FLJ16482 fis, clone BRTHA2017972, highly similar to Mothers against decapentaplegic homolog 7 (SMAD 7)	1.464308135	1.47E-05	down
**PRF1**	hsa:5551	Perforin-1	1.475706332	1.81E-05	down
**GFOD1**	hsa:54438	Glucose-fructose oxidoreductase domain-containing protein 1	1.246649994	2.32E-05	down
**PTGDR**	hsa:5729	Prostaglandin D2 receptor	1.508684656	2.47E-05	down
**GK5**	hsa:256356	Putative glycerol kinase 5	1.241158787	2.78E-05	down
**SGPP1**	hsa:81537	Sphingosine-1-phosphate phosphatase 1	1.359077006	3.05E-05	down
**LOC200772**	hsa:200772	uncharacterized LOC200772	1.331690636	3.72E-05	down
**KLRD1**	hsa:3824	Natural killer cells antigen CD94	1.704893105	4.09E-05	down
**P2RY12**	hsa:64805	cDNA FLJ76055, highly similar to Homo sapiens purinergic receptor P2Y, G-protein coupled, 12 (P2RY12), transcript variant 1, mRNA	1.619781462	4.53E-05	down
**LOC100507625**			1.383686482	4.83E-05	down
**AKR1C3**	hsa:8644	cDNA FLJ58390, highly similar to Aldo-keto reductase family 1 member C3homolog (EC 1.-.-.-)	1.673368339	5.30E-05	down
**YES1**	hsa:7525	Non-specific protein-tyrosine kinase	1.378861195	5.61E-05	down
**TAF15**	hsa:8148	cDNA FLJ53422, highly similar to TATA-binding protein-associated factor 2N	1.369320734	6.91E-05	down

**CBLB**	hsa:868	cDNA FLJ36865 fis, clone ASTRO2016148, highly similar to E3 ubiquitin-protein ligase CBL-B (EC 6.3.2.-)	1.47944548	6.98E-05	down
**TBX21**	hsa:30009	T-box 21 variant	1.462134469	7.24E-05	down
**ENPP4**	hsa:22875	Bis(5'-adenosyl)-triphosphatase ENPP4	1.46138641	9.29E-05	down
**CHST2**	hsa:9435	Carbohydrate sulfotransferase 2	1.33904294	9.38E-05	down
**C9orf38**			1.235254256	1.11E-04	down
**LOC285812**		uncharacterized LOC285812	1.31627647	1.16E-04	down
**LINC00657**	hsa:647979	long intergenic non-protein coding RNA 657	1.280086921	1.23E-04	down
**TRDV3**		cDNA FLJ56790	1.395905876	1.24E-04	down
**PRMT6**	hsa:55170	Protein arginine N-methyltransferase 6	1.472165861	1.43E-04	down
**STAT4**	hsa:6775	cDNA FLJ51337, highly similar to Signal transducer and activator of transcription 4	1.296833665	1.65E-04	down
**EXOSC1**	hsa:51013	Exosome complex component CSL4	1.391265644	1.72E-04	down
**ZNF302**	hsa:55900	cDNA FLJ16476 fis, clone BRSSN2017530, highly similar to Homo sapiens zinc finger protein 302 (ZNF302), transcript variant 1, mRNA	1.296116384	1.86E-04	down
**SSBP3-AS1**	hsa:619518	Putative uncharacterized protein SSBP3-AS1	1.227907195	1.86E-04	down
**LOC100288673**			1.248708154	2.11E-04	down
**SH2D1B**	hsa:117157	SH2 domain-containing protein 1B	1.43397996	2.15E-04	down
**XPO1**	hsa:7514	cDNA FLJ42779 fis, clone BRAWH3005300, highly similar to Exportin-1	1.290130399	2.16E-04	down
**THUMPD3-AS1**	hsa:440944	THUMPD3 antisense RNA 1	1.253221153	2.21E-04	down
**GOLGA4**	hsa:2803	Centrosome-related protein F46	1.237283131	2.21E-04	down
**TGFBR3**	hsa:7049	Transforming growth factor beta receptor type 3	1.516052921	2.23E-04	down
**LRCH3**	hsa:84859	Leucine-rich repeat and calponin homology domain-containing protein 3	1.321497573	2.48E-04	down
**MDM1**	hsa:56890	cDNA, FLJ95264, highly similar to Homo sapiens Mdm4, transformed 3T3 cell double minute 1, p53 binding protein (mouse) (MDM1), transcript variant 2, mRNA	1.246522577	2.57E-04	down
**PLGLB1**	hsa:5343	plasminogen-like B1	1.452476866	2.64E-04	down
**FAM76B**	hsa:143684	Protein FAM76B	1.20038519	2.69E-04	down
**NASP**	hsa:4678	cDNA FLJ53176, highly similar to Nuclear autoantigenic sperm protein	1.246598265	2.80E-04	down
**LOC100507237**			1.229464522	2.97E-04	down
**GOLGA8B**	hsa:440270	Golgin subfamily A member 8B	1.278666449	2.98E-04	down
**ND6**	hsa:4541	NADH-ubiquinone oxidoreductase chain 6	1.404486626	3.02E-04	down
**ENPP5**	hsa:59084	cDNA FLJ76474, highly similar to Homo sapiens ectonucleotide pyrophosphatase/phosphodiesterase 5 (putative function) (ENPP5), mRNA	1.429224718	3.27E-04	down
**RPS24**	hsa:6229	40S ribosomal protein S24	1.358495807	3.40E-04	down

**ARL4C**	hsa:10123	ADP-ribosylation factor-like 4C, isoform CRA_a	1.295112161	3.49E-04	down
**ND2**	hsa:4536	NADH-ubiquinone oxidoreductase chain 2	1.21939174	3.61E-04	down
**PYHIN1**	hsa:149628	Pyrin and HIN domain-containing protein 1	1.345377721	3.61E-04	down
**LOC100128868**			1.267297304	3.65E-04	down
**B4GALT6**	hsa:9331	cDNA, FLJ95759, highly similar to Homo sapiens UDP-Gal:betaGlcNAc beta 1,4-galactosyltransferase, polypeptide 6 (B4GALT6), mRNA	1.258293762	3.68E-04	down
**ADAMTS1**	hsa:9510	cDNA, FLJ95281, highly similar to Homo sapiens a disintegrin-like and metalloprotease (reprolysintype) with thrombospondin type 1 motif, 1 (ADAMTS1), mRNA	1.226686484	3.92E-04	down
**PAPD4**	hsa:167153	Poly(A) RNA polymerase GLD2	1.27016601	3.93E-04	down
**RAP1B**	hsa:5908	RAP1B, member of RAS oncogene family, isoform CRA_a	1.21632751	3.98E-04	down
**SDPR**	hsa:8436	Serum deprivation-response protein	1.355414868	4.07E-04	down
**MEIS1**	hsa:4211	cDNA, FLJ95861, highly similar to Homo sapiens Meis1, myeloid ecotropic viral integration site 1homolog (mouse) (MEIS1), mRNA	1.338750382	4.25E-04	down
**SH2D1A**	hsa:4068	SH2 domain-containing protein 1A	1.247909749	4.25E-04	down
**ARMCX1**	hsa:51309	Armadillo repeat-containing X-linked protein 1	1.265368288	4.49E-04	down
**NKG7**	hsa:4818	Protein NKG7	1.515481482	4.57E-04	down
**RBM14-RBM4**	hsa:100526737	Protein RBM14-RBM4	1.21815143	4.63E-04	down
**ZNF451**	hsa:26036	Zinc finger protein 451	1.237442616	4.88E-04	down
**PDE3B**	hsa:5140	Phosphodiesterase 3B, cGMP-inhibited	1.24877498	5.15E-04	down
**SLAMF7**	hsa:57823	cDNA FLJ76627	1.366469046	5.46E-04	down
**GGTA1P**	hsa:2681	Inactive N-acetyllactosaminide lpha-1,3-galactosyltransferase	1.524089929	5.69E-04	down
**LOC100190986**	hsa:100190986	uncharacterized LOC100190986	1.383901177	5.74E-04	down
**TRA2A**	hsa:29896	Transformer-2 protein homolog alpha	1.240637648	5.76E-04	down
**RBM39**	hsa:9584	cDNA FLJ44170 fis, clone THYMU2035319, highly similar to RNA-binding region-containing protein 2	1.304556893	5.82E-04	down
**AGAP1**	hsa:116987	Arf-GAP with GTPase, ANK repeat and PH domain-containing protein 1	1.339897255	5.91E-04	down
**PNMA1**	hsa:9240	Paraneoplastic antigen Ma1	1.262555358	6.40E-04	down
**CASP8**	hsa:841	HCG16983, isoform CRA_b	1.248404364	6.42E-04	down
**GOLGA8A**	hsa:23015	GOLGA8A protein	1.375485513	6.54E-04	down
**GZMA**	hsa:3001	Granzyme A	1.479486566	6.70E-04	down
**CLIC3**	hsa:9022	Chloride intracellular channel protein 3	1.407413523	6.83E-04	down
**LAX1**	hsa:54900	cDNA FLJ57008, highly similar to Lymphocyte transmembrane adapter 1	1.26153568	6.98E-04	down
**NKTR**	hsa:4820	Natural killer-tumor recognition sequence, isoform CRA_a	1.397790425	7.11E-04	down

**CCL4**	hsa:6351	C-C motif chemokine 4	1.380915621	7.29E-04	down
**RAB27B**	hsa:5874	Ras-related protein Rab-27B	1.418032605	7.47E-04	down
**GCH1**	hsa:2643	GTP cyclohydrolase 1 (Dopa-responsive dystonia), isoform CRA_a	1.269823974	7.53E-04	down
**GBP4**	hsa:115361	cDNA FLJ51459, highly similar to Guanylate-binding protein 4	1.360558893	7.61E-04	down
**SYNJ2BP**	hsa:55333	Synaptojanin 2 binding protein, isoform CRA_a	1.350747477	7.68E-04	down
**LOC283357**		uncharacterized LOC283357	1.308267501	7.86E-04	down
**RNFT1**	hsa:51136	RING finger and transmembrane domain-containing protein1	1.213074234	8.12E-04	down
**SPON2**	hsa:10417	Spondin-2	1.358766962	9.02E-04	down
**GSPT1**	hsa:2935	cDNA FLJ78119, highly similar to Homo sapiens G1 to S phase transition 1 (GSPT1), mRNA	1.24384239	9.37E-04	down
**BCOR**	hsa:54880	cDNA FLJ38041 fis, clone CTONG2013986, highly similar to BCoR protein	1.271405487	9.42E-04	down
**DTHD1**	hsa:401124	Death domain-containing protein 1	1.465308134	0.0010027	down
**SRSF1**	hsa:6426	cDNA FLJ77645, highly similar to Homo sapiens splicing factor, arginine/serine-rich 1 (splicing factor 2, alternate splicing factor) (SFRS1), mRNA	1.241110814	0.001015	down
**MS4A14**	hsa:84689	Membrane-spanning 4-domains subfamily A member 14	1.354853306	0.0010227	down
**CDKN1C**	hsa:1028	Cyclin-dependent kinase inhibitor 1C transcript variant 3	1.368228253	0.001033	down
**GNLY**	hsa:10578	Granulysin	1.48209614	0.0010533	down
**GATAD1**	hsa:57798	GATA zinc finger domain-containing protein 1	1.217534868	0.0010556	down
**PRKCH**	hsa:5583	cDNA FLJ57605, highly similar to Protein kinase C eta type (EC 2.7.11.13)	1.230114277	0.0010677	down
**MAP3K7CL**	hsa:56911	MAP3K7 C-terminal-like protein	1.431043805	0.0010717	down
**PDGFD**	hsa:80310	Platelet-derived growth factor D	1.330733761	0.0011425	down
**GPR180**	hsa:160897	Putative uncharacterized protein DKFZp686I0613	1.200522474	0.0011469	down
**NT5C3A**	hsa:51251	5'-nucleotidase, cytosolic III	1.257968517	0.0011634	down
**C4orf46**	hsa:201725	chromosome 4 open reading frame 46	1.221727768	0.0012104	down
**PRSS23**	hsa:11098	cDNA PSEC0048 fis, clone NT2RP2000028, highly similar to Serine protease 23	1.48149446	0.0012308	down
**ZNF264**	hsa:9422	Zinc finger protein 264, isoform CRA_a	1.247025016	0.0012589	down
**GSE1**	hsa:23199	Genetic suppressor element 1	1.251400315	0.0012784	down
**KBTBD6**	hsa:89890	Kelch repeat and BTB domain-containing protein 6	1.293647626	0.0013902	down
**MIER3**	hsa:166968	Mesoderm induction early response protein 3	1.233777244	0.0014019	down
**CRIM1**	hsa:51232	Cysteine-rich motor neuron 1 protein	1.328246748	0.0014331	down
**ADRB2**	hsa:154	Beta-2 adrenergic receptor	1.30512635	0.0014854	down
**SERPINB6**	hsa:5269	Serpin peptidase inhibitor, clade B (Ovalbumin), member 6, isoform CRA_b	1.216294271	0.0014865	down
**CDK13**	hsa:8621	Cell division cycle 2-like 5 (Cholinesterase-related cell division controller), isoform CRA_b	1.25873012	0.0014948	down
**NUB1**	hsa:51667	NEDD8 ultimate buster 1	1.315427363	0.0015716	down

**NAPEPLD**	hsa:222236	N-acyl-phosphatidylethanolamine-hydrolyzing phospholipase D, isoform CRA_a	1.200000317	0.0016352	down
**DUSP5**	hsa:1847	Dual specificity protein phosphatase 5	1.311374776	0.0016607	down
**PRKACB**	hsa:5567	cAMP-dependent protein kinase catalytic subunit beta isoform 2	1.363776728	0.0016843	down
**FGFBP2**	hsa:83888	Fibroblast growth factor-binding protein 2	1.562044436	0.0017182	down
**STON2**	hsa:85439	Stonin-2	1.251444859	0.0017272	down
**TRMT13**	hsa:54482	cDNA FLJ78543	1.333465555	0.0017553	down
**TUBB1**	hsa:81027	Tubulin beta-1 chain	1.410268804	0.0017592	down
**TXK**	hsa:7294	cDNA FLJ59978, highly similar to Tyrosine-protein kinase TXK (EC 2.7.10.2)	1.355621747	0.0018166	down
**ARL13B**	hsa:200894	cDNA FLJ55982, highly similar to Homo sapiens ADP-ribosylation factor-like 2-like 1 (ARL2L1), transcript variant 1, mRNA	1.205012842	0.0019297	down
**HNRNPA2B1**	hsa:3181	Heterogeneous nuclear ribonucleoprotein A2/B1, isoform CRA_b	1.240947247	0.0019418	down
**SCML1**	hsa:6322	Sex comb on midleg-like protein 1	1.263314206	0.0019837	down
**ATG14**	hsa:22863	Beclin 1-associated autophagy-related key regulator	1.213635093	0.00199	down
**CCL5**	hsa:6352	C-C motif chemokine 5	1.369723291	0.0020875	down
**KIAA1377**	hsa:57562	Uncharacterized protein KIAA1377	1.207201842	0.0020988	down
**PDK4**	hsa:5166	Pyruvate dehydrogenase kinase, isoenzyme 4	1.225538634	0.0021012	down
**PRPF4B**	hsa:8899	PRP4 pre-mRNA processing factor 4 homolog B (Yeast), isoform CRA_a	1.222981363	0.0021049	down
**SLC30A7**	hsa:148867	Zinc transporter 7	1.238430446	0.0021163	down
**KLRK1**	hsa:22914	NKG2-D type II integral membrane protein	1.331415606	0.0021481	down
**MFSD6**	hsa:54842	cDNA FLJ52807	1.214761462	0.0021677	down
**TRAF3IP3**	hsa:80342	TRAF3-interacting JNK-activating modulator	1.396264135	0.0021785	down
**GOLT1B**	hsa:51026	Vesicle transport protein GOT1B	1.2090412	0.0022396	down
**SYTL2**	hsa:54843	Synaptotagmin-like 2, isoform CRA_b	1.418801149	0.0022419	down
**FAM122B**	hsa:159090	Synoviocyte proliferation associated in collagen-induced arthritis 2	1.219530113	0.0022449	down
**SOS1-IT1**		SOS1 intronic transcript 1 (non-protein coding)	1.202184468	0.0022575	down
**HCFC2**	hsa:29915	Host cell factor 2	1.20717322	0.0022581	down
**KLRC4**	hsa:8302	NKG2-F type II integral membrane protein	1.690285608	0.002344	down
**ZNF566**	hsa:84924	cDNA FLJ52734, highly similar to Zinc finger protein 566	1.328611915	0.0023447	down
**AGAP4**	hsa:119016	Arf-GAP with GTPase, ANK repeat and PH domain-containing protein 4	1.25835455	0.0024093	down
**PGRMC1**	hsa:10857	Membrane-associated progesterone receptor component 1	1.263580915	0.0024602	down
**GBP5**	hsa:115362	Guanylate-binding protein 5	1.441397515	0.0024814	down
**RGS18**	hsa:64407	cDNA FLJ78111, highly similar to Homo sapiens regulator of G-protein signalling 18 (RGS18), mRNA	1.289551178	0.0025296	down

**KLRC2**	hsa:3822	NKG2-C type II integral membrane protein	1.537120503	0.0025814	down
**SYTL3**	hsa:94120	cDNA FLJ61334, moderately similar to Synaptotagmin-like protein 3	1.283321402	0.0025893	down
**MED21**	hsa:9412	SRB7 suppressor of RNA polymerase B homolog (Yeast), isoform CRA_b	1.244328269	0.0025966	down
**PTPN22**	hsa:26191	Tyrosine-protein phosphatase non-receptor type 22	1.211097494	0.0025968	down
**TARDBP**	hsa:23435	TAR DNA binding protein, isoform CRA_b	1.250612767	0.0026061	down
**ZNF431**	hsa:170959	Zinc finger protein 431, isoform CRA_b	1.332166741	0.0026131	down
**INAFM2**	hsa:100505573	Putative transmembrane protein INAFM2	1.232634124	0.0026368	down
**ZNF708**	hsa:7562	Zinc finger protein 708	1.283616171	0.0026577	down
**MLC1**	hsa:23209	Megalencephalic leukoencephalopathy with subcortical cysts 1, isoform CRA_a	1.203477487	0.0027077	down
**FNBP4**	hsa:23360	Formin-binding protein 4	1.253378549	0.0028087	down
**MOB1B**	hsa:92597	cDNA FLJ36226 fis, clone THYMU2001018, highly similar to Mps one binder kinase activator-like 1A	1.222842681	0.0028119	down
**IGF2BP3**	hsa:10643	Insulin-like growth factor 2 mRNA-binding protein 3	1.323701925	0.002814	down
**WDR11**	hsa:55717	cDNA, FLJ96114, highly similar to Homo sapiens bromodomain and WD repeat domain containing 2 (BRWD2), mRNA	1.274659613	0.002876	down
**PARP15**	hsa:165631	cDNA FLJ40196 fis, clone TESTI2019794, highly similar to Homo sapiens poly (ADP-ribose) polymerase family, member 15 (PARP15), mRNA	1.214374341	0.0028957	down
**DCLRE1A**	hsa:9937	DNA cross-link repair 1A protein	1.22321658	0.0029039	down
**NHLRC3**	hsa:387921	NHL repeat-containing protein 3	1.231887015	0.0029219	down
**METTL3**	hsa:56339	cDNA FLJ36190 fis, clone TESTI2027271, highly similar to N6-adenosine-methyltransferase 70 kDa subunit (EC 2.1.1.62)	1.200102253	0.0029532	down
**FAM169A**	hsa:26049	Soluble lamin-associated protein of 75 kDa	1.291705317	0.0030112	down
**SDCCAG3**	hsa:10807	Serologically defined colon cancer antigen 3	1.215732202	0.003075	down
**ZNF367**	hsa:195828	Zinc finger protein 367	1.27243673	0.0030783	down
**ITGB3BP**	hsa:23421	Integrin beta 3 binding protein (Beta3-endonexin), isoform CRA_a	1.286375381	0.0031025	down
**TMEM45A**	hsa:55076	Transmembrane protein 45A, isoform CRA_a	1.537942249	0.0031141	down
**PLGLA**	hsa:285189	Plasminogen-like protein A	1.432903494	0.0031592	down
**LOC100506395**			1.306368158	0.0031984	down
**C15orf54**	hsa:400360	chromosome 15 open reading frame 54	1.373115988	0.0032844	down
**GIMAP6**	hsa:474344	cDNA, FLJ95514, highly similar to Homo sapiens human immune associated nucleotide 2 (hIAN2), mRNA	1.254215949	0.0033297	down
**SLC38A9**	hsa:153129	Uncharacterized protein	1.225260965	0.0033327	down
**XCL2**	hsa:6846	Cytokine SCM-1 beta	1.376176795	0.0033444	down

**JAKMIP2**	hsa:9832	JAKMIP2 protein	1.203522515	0.0034349	down
**ADAM1A**	hsa:8759	ADAM metallopeptidase domain 1A, pseudogene	1.305948646	0.0034862	down
**XCL1**	hsa:6375	Lymphotactin	1.21629634	0.0036217	down
**PKD2**	hsa:5311	cDNA FLJ50446, highly similar to Polycystin-2	1.292201479	0.0036425	down
**LOC100506748**			1.248487831	0.0036845	down
**PTPN4**	hsa:5775	Tyrosine-protein phosphatase non-receptor type 4	1.293067088	0.0038188	down
**EIF4A2**	hsa:1974	cDNA FLJ58834, highly similar to Eukaryotic initiation factor 4A-II (EC 3.6.1.-)	1.25367461	0.0038818	down
**THAP9-AS1**	hsa:100499177	THAP9 antisense RNA 1	1.237802113	0.0040032	down
**HNRNPH1**	hsa:3187	cDNA FLJ54533, highly similar to Heterogeneous nuclear ribonucleoprotein H	1.263293027	0.004048	down
**GVINP1**	hsa:387751	Interferon-induced very large GTPase 1	1.26713917	0.0040797	down
**ZNF567**	hsa:163081	cDNA, FLJ94224	1.266137627	0.0041006	down
**MTR**	hsa:4548	cDNA FLJ53729, moderately similar to Methionine synthase (EC 2.1.1.13)	1.231910022	0.004145	down
**MBNL2**	hsa:10150	MBNL2 protein	1.233900244	0.0041479	down
**CXCL5**	hsa:6374	C-X-C motif chemokine 5	1.577233037	0.0041597	down
**HNRNPU-AS1**	hsa:284702	HNRNPU antisense RNA 1	1.25426582	0.004228	down
**TRIM73**	hsa:375593	Tripartite motif-containing protein 73	1.313078518	0.004312	down
**C5orf24**	hsa:134553	chromosome 5 open reading frame 24	1.20717662	0.0043436	down
**GTF3C3**	hsa:9330	General transcription factor 3C polypeptide 3	1.261796928	0.0044759	down
**TXNL1**	hsa:9352	cDNA, FLJ94230, highly similar to Homo sapiens thioredoxin-like 1 (TXNL1), mRNA	1.21579065	0.0044877	down
**NLRC3**	hsa:197358	NOD3 protein, isoform CRA_c	1.258218791	0.0045674	down
**CD226**	hsa:10666	CD226 antigen	1.28587013	0.0046042	down
**CDKN2AIP**	hsa:55602	cDNA FLJ38861 fis, clone MESAN2011984, highly similar to Homo sapiens collaborates/cooperates with ARF (alternate reading frame) protein (CARF), mRNA	1.214001762	0.0046054	down
**CX3CR1**	hsa:1524	CX3C chemokine receptor 1	1.246772815	0.004772	down
**ZBTB6**	hsa:10773	Zinc finger and BTB domain-containing protein 6	1.351388875	0.0048068	down
**HERC2P9**	hsa:440248	hect domain and RLD 2 pseudogene 9	1.213990485	0.0048108	down
**ANKRD36B**	hsa:57730	ankyrin repeat domain 36B	1.375789456	0.0048683	down
**ZNF304**	hsa:57343	Zinc finger protein 304, isoform CRA_a	1.285344295	0.0048937	down
**MIR186**	hsa:406962	microRNA 186	1.253861101	0.0049299	down
**LOC158402**		uncharacterized LOC158402	1.23775567	0.0049636	down
**NDUFAF7**	hsa:55471	NADH dehydrogenase [ubiquinone] complex I, assembly factor 7	1.227682646	0.0049926	down
**TMEM128**	hsa:85013	Transmembrane protein 128, isoform CRA_b	1.21548238	0.0050851	down
**CHD9**	hsa:80205	cDNA FLJ57565, highly similar to Chromodomain-helicase-DNA-binding protein 9 (EC 3.6.1.-)	1.214632662	0.0051903	down

**KLRG1**	hsa:10219	cDNA FLJ56726, highly similar to Homo sapiens killer cell lectin-like receptor subfamily G, member 1 (KLRG1), mRNA	1.338115587	0.0052081	down
**HMG20A**	hsa:10363	cDNA, FLJ79180, highly similar to High mobility group protein 20A	1.250243213	0.0052142	down
**ZNF217**	hsa:7764	cDNA FLJ77047, highly similar to Homo sapiens zinc finger protein 217 (ZNF217), mRNA	1.21924904	0.0053269	down
**MIS12**	hsa:79003	cDNA FLJ76754, highly similar to Homo sapiens MIS12 homolog (yeast) (MIS12), mRNA	1.240335074	0.0053373	down
**PSIP1**	hsa:11168	PC4 and SFRS1-interacting protein	1.236071836	0.0053779	down
**MPP5**	hsa:64398	MAGUK p55 subfamily member 5	1.206889077	0.0054772	down
**VPS13C**	hsa:54832	Vacuolar protein sorting-associated protein 13C	1.242782485	0.0055227	down
**CMPK1**	hsa:51727	UMP-CMP kinase	1.215242331	0.0056325	down
**RUNX3**	hsa:864	Runt-related transcription factor 3, isoform CRA_b	1.209578172	0.0056931	down
**C3orf38**	hsa:285237	Uncharacterized protein C3orf38	1.200799249	0.0057932	down
**SPIN4**	hsa:139886	Spindlin-4	1.244200889	0.0058126	down
**NET1**	hsa:10276	cDNA FLJ54782, highly similar to Neuroepithelial cell-transforming gene 1protein	1.239222182	0.0059909	down
**LINS**	hsa:55180	lines homolog (Drosophila)	1.216429112	0.0060798	down
**CRYZL1**	hsa:9946	Quinone oxidoreductase-like protein 1	1.220750812	0.0060959	down
**PPBP**	hsa:5473	Platelet basic protein	1.262810324	0.0062069	down
**GRB14**	hsa:2888	cDNA, FLJ93646, highly similar to Homo sapiens growth factor receptor-bound protein 14 (GRB14), mRNA	1.396473131	0.0062329	down
**ZNF37BP**	hsa:100129482	zinc finger protein 37B, pseudogene	1.274638233	0.0063995	down
**MRPL19**	hsa:9801	39S ribosomal protein L19, mitochondrial	1.261324588	0.0064087	down
**OST4**	hsa:100128731	Dolichyl-diphosphooligosaccharide--protein glycosyltransferase subunit 4	1.207042195	0.006648	down
**LOC727820**		uncharacterized LOC727820	1.207544177	0.0068079	down
**ZNF655**	hsa:79027	cDNA FLJ16616 fis, clone TESTI4013597, highly similar to Zinc finger protein 655	1.237976039	0.0068372	down
**RNF213**	hsa:57674	Chromosome 17 open reading frame 27, isoform CRA_a	1.238402864	0.0069118	down
**EHD3**	hsa:30845	EH domain-containing protein 3	1.273380635	0.006993	down
**GBP3**	hsa:2635	Guanylate-binding protein 3	1.294155075	0.0069948	down
**CLHC1**	hsa:130162	clathrin heavy chain linker domain containing 1	1.275293667	0.0070475	down
**PRMT2**	hsa:3275	Protein arginine N-methyltransferase 2	1.247989042	0.0071075	down
**RBM3**	hsa:5935	RNA binding motif (RNP1, RRM) protein 3, isoform CRA_c	1.20429289	0.0071151	down
**TMX3**	hsa:54495	Protein disulfide-isomerase TMX3	1.201166308	0.0071313	down
**NNT-AS1**	hsa:100652772	NNT antisense RNA 1	1.325903618	0.0072897	down
**PPA2**	hsa:27068	cDNA FLJ76209, highly similar to Homo sapiens inorganic pyrophosphatase 2 (PPA2), transcript variant 2, mRNA	1.24291123	0.0072907	down

**TRAM1**	hsa:23471	Translocating chain-associated membrane protein 1	1.20335798	0.0073206	down
**PWAR5**	hsa:8123	Prader Willi/Angelman region RNA 5	1.245173873	0.0074367	down
**FMNL2**	hsa:114793	cDNA FLJ37546 fis, clone BRCAN2027364, highly similar to Homo sapiens formin-like 2 (FMNL2), transcript variant 2, mRNA	1.244321245	0.007539	down
**CEP104**	hsa:9731	Glycine-, glutamate-, thienylcyclohexylpiperidine-binding protein, isoform CRA_c	1.244853829	0.0076782	down
**GIMAP2**	hsa:26157	Immunity associated protein 2	1.238869897	0.0078151	down
**ZNF600**	hsa:162966	Zinc finger protein 600	1.354093778	0.0079161	down
**KLRC3**	hsa:3823	NKG2-E type II integral membrane protein	1.453486174	0.0079188	down
**ALDH1A1**	hsa:216	cDNA FLJ51786, highly similar to Retinal dehydrogenase 1 (EC 1.2.1.36)	1.339116441	0.00822	down
**ZNF548**	hsa:147694	cDNA FLJ58345, highly similar to Zinc finger protein 548	1.259775072	0.0082376	down
**MFSD8**	hsa:256471	cDNA FLJ50485	1.206937546	0.0084532	down
**RTP4**	hsa:64108	Receptor-transporting protein 4	1.233480117	0.0084838	down
**ANKRD46**	hsa:157567	Ankyrin repeat domain-containing protein 46	1.232207189	0.0085499	down
**FARSB**	hsa:10056	cDNA FLJ75460, highly similar to Homo sapiens phenylalanine-tRNA synthetase-like, beta subunit, mRNA	1.290347837	0.0086111	down
**ALG10B**	hsa:144245	Putative Dol-P-Glc:Glc(2)Man(9)GlcNAc(2)-PP-Dol alpha-1,2-glucosyltransferase	1.234984621	0.0090425	down
**PDS5B**	hsa:23047	Sister chromatid cohesion protein PDS5 homolog B	1.20080612	0.0092351	down
**PROSER2**	hsa:254427	proline and serine rich 2	1.218415522	0.0093119	down
**LOC100507486**			1.247222739	0.009381	down
**SLC4A4**	hsa:8671	Solute carrier family 4, sodium bicarbonate cotransporter, member 4, isoform CRA_d	1.226978237	0.009442	down
**C2orf88**	hsa:84281	Small membrane A-kinase anchor protein	1.288972717	0.0094783	down
**LOC100288656**		ankyrin repeat domain 20 family, member A2 pseudogene	1.340219679	0.0095666	down
**ZFAND1**	hsa:79752	AN1-type zinc finger protein 1	1.205605283	0.0096101	down
**NR2C2**	hsa:7182	Nuclear receptor subfamily 2 group C member 2	1.206644503	0.0096431	down
**APOL6**	hsa:80830	Apolipoprotein L6	1.209153415	0.0096692	down
**UBE2Q2**	hsa:92912	Ubiquitin-conjugating enzyme E2 Q2	1.223389435	0.009674	down
**HGD**	hsa:3081	cDNA FLJ42297 fis, clone TLIVE2009087, highly similar to HOMOGENTISATE 1,2-DIOXYGENASE (EC 1.13.11.5)	1.277571741	0.0097884	down
**GUCY1B3**	hsa:2983	cDNA, FLJ96185, highly similar to Homo sapiens guanylate cyclase 1, soluble, beta 3 (GUCY1B3), mRNA	1.362848635	0.0098066	down
**ACRBP**	hsa:84519	Acrosin-binding protein	1.298430669	0.0099642	down
**RRN3**	hsa:54700	cDNA FLJ56083, highly similar to RNA polymerase I-specific transcriptioninitiation factor RRN3	1.220492999	0.0100356	down

**RHOBTB1**	hsa:9886	Rho-related BTB domain containing 1, isoform CRA_c	1.432544549	0.0100406	down
**PRKAA1**	hsa:5562	5'-AMP-activated protein kinase catalytic subunit alpha-1	1.26642509	0.0102746	down
**RBM43**	hsa:375287	RNA-binding protein 43	1.221027521	0.0102858	down
**SIGLEC17P**	hsa:284367	sialic acid binding Ig-like lectin 17, pseudogene	1.250534701	0.0103224	down
**HAUS6**	hsa:54801	HAUS augmin-like complex subunit 6	1.230120635	0.0105522	down
**PRKAR2B**	hsa:5577	Protein kinase, cAMP-dependent, regulatory, type II, beta, isoform CRA_a	1.352778359	0.0107749	down
**KIN**	hsa:22944	DNA/RNA-binding protein KIN17	1.201746452	0.0111266	down
**S1PR1**	hsa:1901	cDNA FLJ58121, highly similar to Sphingosine 1-phosphate receptor Edg-1	1.293688229	0.0112073	down
**CDC37L1**	hsa:55664	Hsp90 co-chaperone Cdc37-like 1	1.203936577	0.0112723	down
**MLH3**	hsa:27030	MutL homolog 3 (E. coli), isoform CRA_b	1.329932644	0.0113217	down
**MPHOSPH9**	hsa:10198	M-phase phosphoprotein 9	1.226007117	0.0113541	down
**LOC100510542**			1.200223781	0.0114408	down
**KDELC2**	hsa:143888	KDEL motif-containing protein 2	1.211100705	0.0114483	down
**TCF7L2**	hsa:6934	cDNA FLJ90224 fis, clone NT2RM1000789, highly similar to Mus musculus transcription factor 7-like 2, T-cell specific, HMG-box (Tcf7l2), mRNA	1.266842745	0.0115308	down
**C1orf109**	hsa:54955	chromosome 1 open reading frame 109	1.230273298	0.0115312	down
**OFD1**	hsa:8481	Oral-facial-digital syndrome 1 protein	1.231288042	0.0118217	down
**ALG13**	hsa:79868	Putative bifunctional UDP-N-acetylglucosamine transferase and deubiquitinase ALG13	1.211768082	0.0119262	down
**SCAF4**	hsa:57466	Splicing factor, arginine/serine-rich 15	1.210569772	0.0119301	down
**A2M-AS1**	hsa:144571	A2M antisense RNA 1 (head to head)	1.417768003	0.0121371	down
**ABCB1**	hsa:5243	ATP-binding cassette, sub-family B (MDR/TAP), member 1	1.260718013	0.0124007	down
**HLA-DPA1**	hsa:3113	MHC class II antigen	1.238914475	0.0127216	down
**TMED2**	hsa:10959	cDNA FLJ52153, highly similar to Transmembrane emp24 domain-containing protein 2	1.234988287	0.0130326	down
**TARP**	hsa:445347	TCR gamma alternate reading frame protein	1.416552752	0.0132136	down
**GOLGA6L9**	hsa:440295	golgin A6 family-like 9	1.320244007	0.0132354	down
**TMEM40**	hsa:55287	cDNA FLJ55821, highly similar to Homo sapiens transmembrane protein 40 (TMEM40), mRNA	1.244547589	0.0134494	down
**SIDT1**	hsa:54847	cDNA FLJ52305, highly similar to SID1 transmembrane family member 1	1.227029033	0.0135275	down
**PAXIP1-AS1**	hsa:202781	PAXIP1 antisense RNA 1 (head to head)	1.209146768	0.0136045	down
**ATL1**	hsa:51062	Atlastin-1	1.211187887	0.0139377	down
**EPS8**	hsa:2059	cDNA FLJ56405, highly similar to Epidermal growth factor receptor kinase substrate 8	1.322058812	0.0140736	down
**MBTD1**	hsa:54799	MBT domain-containing protein 1	1.214950749	0.0143443	down
**RBAK**	hsa:57786	RB-associated KRAB zinc finger protein	1.28409178	0.0143846	down
**GBP1**	hsa:2633	cDNA FLJ51602, highly similar to Interferon-induced guanylate-binding protein 1	1.306054448	0.0145587	down

**SOS1**	hsa:6654	Son of sevenless homolog 1 (Drosophila), isoform CRA_d	1.202338191	0.0146784	down
**MINPP1**	hsa:9562	cDNA, FLJ93389, highly similar to Homo sapiens multiple inositol polyphosphate histidine phosphatase, 1 (MINPP1), mRNA	1.202510212	0.0147664	down
**GNPDA2**	hsa:132789	Glucosamine-6-phosphate isomerase 2	1.215439809	0.0151724	down
**CCNJ**	hsa:54619	cDNA FLJ35041 fis, clone OCBBF2017458, highly similar to Homo sapiens cyclin J (CCNJ), mRNA	1.203061369	0.0152461	down
**LOC374443**	hsa:374443	C-type lectin domain family 2, member D pseudogene	1.24570738	0.0153306	down
**GPR18**	hsa:2841	G protein-coupled receptor 18	1.368094034	0.0154065	down
**STAT1**	hsa:6772	Uncharacterized protein	1.215638434	0.0158167	down
**RBM12B**	hsa:389677	RNA-binding protein 12B	1.237556448	0.0160693	down
**HLTF**	hsa:6596	cDNA FLJ76830, highly similar to Homo sapiens SWI/SNF related, matrix associated, actin dependent regulator of chromatin, subfamily a, member 3 (SMARCA3), transcript variant 1, mRNA	1.20420342	0.0162193	down
**ASAP2**	hsa:8853	Arf-GAP with SH3 domain, ANK repeat and PH domain-containing protein2	1.297043436	0.0162784	down
**KPNA5**	hsa:3841	Importin subunit alpha-6	1.273386211	0.0166855	down
**CA2**	hsa:760	Carbonic anhydrase 2	1.308012405	0.016782	down
**STK39**	hsa:27347	STE20/SPS1-related proline-alanine-rich protein kinase	1.237566314	0.0171743	down
**CEP76**	hsa:79959	Centrosomal protein of 76 kDa	1.21131721	0.0174125	down
**NAP1L3**	hsa:4675	cDNA FLJ33453 fis, clone BRAMY2000181, highly similar to NUCLEOSOME ASSEMBLY PROTEIN 1-LIKE 3	1.338585818	0.0175009	down
**AHSA2**	hsa:130872	Activator of 90 kDa heat shock protein ATPase homolog 2	1.392046374	0.017549	down
**FZD6**	hsa:8323	Frizzled homolog 6 (Drosophila), isoform CRA_a	1.307264084	0.0176893	down
**PNN**	hsa:5411	Pinin	1.21436172	0.0177259	down
**SHPRH**	hsa:257218	cDNA FLJ45012 fis, clone BRAWH3013264, highly similar to Homo sapiens SNF2 histone linker PHD RING helicase (SHPRH), mRNA	1.215576379	0.0178658	down
**USP34**	hsa:9736	Ubiquitin carboxyl-terminal hydrolase 34	1.209987131	0.0182295	down
**GPR174**	hsa:84636	Probable G-protein coupled receptor 174	1.238313419	0.0186565	down
**ZNF623**	hsa:9831	Zinc finger protein 623	1.221275163	0.0186991	down
**LOC100505501**	hsa:100505501	uncharacterized LOC100505501	1.237968456	0.0194415	down
**ANKRD36**	hsa:375248	ankyrin repeat domain 36	1.20987294	0.0197738	down
**MRPL32**	hsa:64983	Mitochondrial ribosomal protein L32	1.209734402	0.019814	down
**TRMT11**	hsa:60487	tRNA (guanine(10)-N2)-methyltransferase homolog	1.229135443	0.019872	down
**KIAA1279**	hsa:26128	KIF1-binding protein	1.20319442	0.0199593	down

**ITM2A**	hsa:9452	Integral membrane protein 2A	1.280749215	0.0200225	down
**ZNF184**	hsa:7738	Zinc finger protein 184 (Kruppel-like), isoform CRA_a	1.229881667	0.0201083	down
**OTUD6B**	hsa:51633	OTU domain-containing protein 6B	1.21227972	0.0202981	down
**TMEM263**	hsa:90488	Transmembrane protein 263	1.247176566	0.0204016	down
**LOC285628**		uncharacterized LOC285628	1.220996747	0.0204431	down
**BEX4**	hsa:56271	Protein BEX4	1.216324412	0.020519	down
**MAP4K3**	hsa:8491	cDNA FLJ11491 fis, clone HEMBA1001921, highly similar to Mitogen-activated protein kinase kinase kinase kinase 3 (EC 2.7.11.1)	1.295302355	0.0206254	down
**GUCY1A3**	hsa:2982	cDNA FLJ39269 fis, clone OCBBF2010420, highly similar to Guanylate cyclase soluble subunit alpha-3 (EC 4.6.1.2)	1.325736825	0.0208094	down
**TNFSF4**	hsa:7292	Tumor necrosis factor (Ligand) superfamily, member 4 (Tax-transcriptionally activated glycoprotein 1, 34kDa), isoform CRA_a	1.264217336	0.021202	down
**NOG**	hsa:9241	cDNA, FLJ94753, highly similar to Homo sapiens noggin (NOG), mRNA	1.401979197	0.0213356	down
**IL15**	hsa:3600	Interleukin-15	1.233607933	0.0217513	down
**LOC441259**		postmeiotic segregation increased 2 pseudogene	1.265736608	0.0222177	down
**ATP1B1**	hsa:481	ATPase, Na+/K+ transporting, beta 1 polypeptide, isoform CRA_a	1.234124887	0.0223378	down
**LOC100505971**			1.213814147	0.0225638	down
**LOC100506245**			1.212358266	0.0226894	down
**HOXB2**	hsa:3212	Homeobox B2, isoform CRA_b	1.208939508	0.0231606	down
**SMAD4**	hsa:4089	Mothers against decapentaplegic homolog 4	1.25856414	0.0232933	down
**ZNF439**	hsa:90594	Zinc finger protein 439	1.265134225	0.023392	down
**PF4V1**	hsa:5197	Platelet factor 4 variant	1.53779448	0.023418	down
**GIMAP7**	hsa:168537	Immune associated nucleotide	1.207636262	0.0237653	down
**BCL11B**	hsa:64919	B-cell lymphoma/leukemia 11B	1.274581993	0.0245403	down
**RPS5**	hsa:6193	Ribosomal protein S5, isoform CRA_a	1.215415525	0.0254082	down
**SPARC**	hsa:6678	cDNA, FLJ96669, highly similar to Homo sapiens secreted protein, acidic, cysteine-rich (osteonectin)(SPARC), mRNA	1.332860545	0.0256566	down
**GTF2H2B**	hsa:653238	GTF2H2C protein	1.249245541	0.0258423	down
**PDIK1L**	hsa:149420	Serine/threonine-protein kinase PDIK1L	1.263065599	0.0259044	down
**CXCL10**	hsa:3627	C-X-C motif chemokine 10	1.27126388	0.0259259	down
**C12orf75**	hsa:387882	chromosome 12 open reading frame 75	1.245143319	0.0263598	down
**RNF11**	hsa:26994	RING finger protein 11	1.218599197	0.0265578	down
**IRAK1BP1**	hsa:134728	Interleukin-1 receptor-associated kinase 1-binding protein 1	1.213203906	0.0266207	down

**TMA16**	hsa:55319	Translation machinery-associated protein 16	1.224349723	0.026817	down
**PPM1K**	hsa:152926	Protein phosphatase 1K, mitochondrial	1.216196928	0.0279541	down
**TMTC3**	hsa:160418	cDNA FLJ52097, weakly similar to Homo sapiens transmembrane and tetratricopeptide repeat containing 1 (TMTC1), mRNA	1.217847092	0.0283196	down
**IFNG**	hsa:3458	Interferon gamma	1.258027643	0.0287894	down
**MTURN**	hsa:222166	Maturin	1.204898388	0.028817	down
**LOC100509911**			1.216238039	0.029196	down
**MRPL50**	hsa:54534	39S ribosomal protein L50, mitochondrial	1.238324005	0.0306485	down
**SMN2**	hsa:6607	Survival motor neuron protein isoform 6B	1.227238473	0.0306819	down
**CHRM3-AS2**	hsa:100506915	CHRM3 antisense RNA 2	1.365536936	0.0307263	down
**MGC12488**		uncharacterized protein MGC12488	1.20540233	0.031063	down
**GP6**	hsa:51206	Platelet glycoprotein VI	1.277935476	0.0310643	down
**TC2N**	hsa:123036	Tandem C2 domains nuclear protein	1.255132807	0.0315493	down
**SACS**	hsa:26278	Sacsin	1.201841012	0.0324	down
**PEAR1**	hsa:375033	Platelet endothelial aggregation receptor 1	1.210359586	0.0324567	down
**SLC25A32**	hsa:81034	Solute carrier family 25, member 32, isoform CRA_a	1.205859771	0.0325391	down
**HCG11**	hsa:493812	HLA complex group 11 (non-protein coding)	1.236203533	0.0327435	down
**GPRASP1**	hsa:9737	G-protein coupled receptor-associated sorting protein 1	1.252867404	0.0329838	down
**KDSR**	hsa:2531	Follicular lymphoma variant translocation 1, isoform CRA_a	1.263331381	0.0330581	down
**ANKRD13C**	hsa:81573	Ankyrin repeat domain-containing protein 13C	1.21465832	0.0358684	down
**HIST1H3H**	hsa:8357	histone cluster 1, H3h	1.270394255	0.0363681	down
**MS4A7**	hsa:58475	Membrane-spanning 4-domains, subfamily A, member 7, isoform CRA_c	1.271415199	0.0372664	down
**ZNF83**	hsa:55769	Zinc finger protein 83 (HPF1), isoform CRA_a	1.204460855	0.0380654	down
**TRPC1**	hsa:7220	TRPC1 splice variant	1.263261756	0.0382562	down
**DDHD2**	hsa:23259	cDNA FLJ31963 fis, clone NT2RP7007610, highly similar to Homo sapiens DDHD domain containing 2 (DDHD2), mRNA	1.222284537	0.0383592	down
**LOC100507397**		uncharacterized LOC100507397	1.236786778	0.0412996	down
**THBS1**	hsa:7057	Thrombospondin 1, isoform CRA_a	1.216590775	0.0423247	down
**NEXN**	hsa:91624	cDNA FLJ55951, highly similar to Homo sapiens nexilin (F actin binding protein) (NEXN), mRNA	1.206935457	0.0429466	down
**SLAIN1**	hsa:122060	SLAIN motif family, member 1	1.259798151	0.0429804	down
**FANCL**	hsa:55120	cDNA FLJ51649, highly similar to Ubiquitin ligase protein FANCL (EC 6.3.2.-)	1.232279876	0.0435979	down
**ZC2HC1A**	hsa:51101	Zinc finger C2HC domain-containing protein 1A	1.232425262	0.044013	down

**GATA3**	hsa:2625	cDNA FLJ56578, highly similar to Trans-acting T-cell-specific transcription factor GATA-3	1.227239651	0.0445615	down
**CRYZ**	hsa:1429	Quinone oxidoreductase	1.247076136	0.045911	down
**RASGRP1**	hsa:10125	cDNA, FLJ94771, highly similar to Homo sapiens RAS guanyl releasing protein 1 (calcium and DAG-regulated) (RASGRP1), mRNA	1.228334736	0.0469995	down
**MASTL**	hsa:84930	Serine/threonine-protein kinase greatwall	1.228720096	0.0471263	down
**SH3BGRL2**	hsa:83699	SH3 domain-binding glutamic acid-rich-like protein 2	1.25552888	0.0475114	down
**LOC388210**	hsa:388210	uncharacterized LOC388210	1.435061444	2.00E-06	up
**RPS11**	hsa:6205	40S ribosomal protein S11	1.236302747	2.03E-06	up
**CNN2**	hsa:1265	cDNA FLJ52495, highly similar to Calponin-2	1.244198647	6.40E-06	up
**S100A8**	hsa:6279	Protein S100-A8	1.240892543	6.71E-06	up
**LOC100506828**			1.510953753	7.91E-06	up
**FOLR1**	hsa:2348	Folate receptor 1 (Adult), isoform CRA_a	1.251703711	9.91E-06	up
**RPL38**	hsa:6169	Ribosomal protein L38, isoform CRA_a	1.302994468	2.28E-05	up
**CEACAM3**	hsa:1084	Carcinoembryonic antigen-related cell adhesion molecule 3	1.251589311	3.20E-05	up
**SHKBP1**	hsa:92799	cDNA, FLJ93157, highly similar to Homo sapiens SH3KBP1 binding protein 1 (SHKBP1), mRNA	1.331164915	5.02E-05	up
**FES**	hsa:2242	Tyrosine-protein kinase Fes/Fps	1.26253808	1.47E-04	up
**ASGR2**	hsa:433	Asialoglycoprotein receptor 2	1.431197551	1.58E-04	up
**ST14**	hsa:6768	Suppressor of tumorigenicity 14 protein homolog	1.214592988	1.85E-04	up
**EMILIN2**	hsa:84034	EMILIN-2	1.205032983	1.95E-04	up
**APCDD1**	hsa:147495	Protein APCDD1	1.218527601	2.73E-04	up
**OMG**	hsa:4974	Oligodendrocyte-myelin glycoprotein	1.372431918	3.50E-04	up
**TMEM88**	hsa:92162	Transmembrane protein 88	1.276552977	3.54E-04	up
**MTPN**	hsa:136319	Myotrophin	1.273371878	3.66E-04	up
**LRRC4**	hsa:64101	Leucine-rich repeat-containing protein 4	1.25932907	3.72E-04	up
**SULT1B1**	hsa:27284	Sulfotransferase family cytosolic 1B member 1	1.433407622	4.21E-04	up
**S100A12**	hsa:6283	Protein S100-A12	1.74256004	5.18E-04	up
**SFN**	hsa:2810	14-3-3 protein sigma	1.291145405	5.49E-04	up
**PIM3**	hsa:415116	PIM3 protein	1.253510275	5.92E-04	up
**LOC101927851**	hsa:101927851	uncharacterized LOC101927851	1.309346892	6.11E-04	up
**DHRS13**	hsa:147015	Dehydrogenase/reductase SDR family member 13	1.357269656	6.22E-04	up
**CA4**	hsa:762	Carbonic anhydrase 4	1.316697079	6.62E-04	up

**TLR5**	hsa:7100	Toll-like receptor 5	1.455642302	8.25E-04	up
**KIAA0319**	hsa:9856	Dyslexia-associated protein KIAA0319	1.213095046	9.38E-04	up
**DYSF**	hsa:8291	cDNA FLJ55344, highly similar to Dysferlin	1.375551823	1.01E-03	up
**SLPI**	hsa:6590	Antileukoproteinase	1.593164814	1.02E-03	up
**GLT1D1**	hsa:144423	cDNA FLJ51476	1.361923039	1.11E-03	up
**IL4R**	hsa:3566	Interleukin 4 receptor alpha	1.293281217	1.19E-03	up
**GPR15**	hsa:2838	cDNA, FLJ93906, highly similar to Homo sapiens G protein-coupled receptor 15 (GPR15), mRNA	1.216719121	1.20E-03	up
**TSHZ3**	hsa:57616	Teashirt zinc finger homeobox 3	1.287326153	1.20E-03	up
**CECR6**	hsa:27439	cat eye syndrome chromosome region, candidate 6	1.210285868	1.73E-03	up
**TLR10**	hsa:81793	Toll-like receptor 10	1.246940727	1.74E-03	up
**ADM**	hsa:133	ADM	1.6266286	1.76E-03	up
**MMP25**	hsa:64386	Matrix metalloproteinase-25	1.218833548	1.90E-03	up
**DSC2**	hsa:1824	cDNA FLJ76245, highly similar to Homo sapiens desmocollin 2 (DSC2), transcript variant Dsc2a, mRNA	1.69300233	2.38E-03	up
**S100A9**	hsa:6280	Protein S100-A9	1.390910943	2.44E-03	up
**ASGR1**	hsa:432	Asialoglycoprotein receptor 1	1.263522369	2.69E-03	up
**INSC**	hsa:387755	Protein inscuteable homolog	1.3805439	2.82E-03	up
**ANXA3**	hsa:306	Annexin A3	1.830528375	2.85E-03	up
**REM2**	hsa:161253	GTP-binding protein REM 2	1.287679471	2.87E-03	up
**DGAT2**	hsa:84649	Diacylglycerol O-acyltransferase 2	1.340359149	3.25E-03	up
**UBE2J1**	hsa:51465	Ubiquitin-conjugating enzyme E2 J1	1.244503138	3.26E-03	up
**RNF19B**	hsa:127544	E3 ubiquitin-protein ligase RNF19B	1.206450836	3.28E-03	up
**LILRA5**	hsa:353514	Leukocyte immunoglobulin-like receptor subfamily A member 5	1.256153766	3.37E-03	up
**ATP6V0D1**	hsa:9114	cDNA, FLJ93507, highly similar to Homo sapiens ATPase, H+ transporting, lysosomal 38kDa, V0 subunit d isoform 1 (ATP6V0D1), mRNA	1.202058458	3.64E-03	up
**PTEN**	hsa:5728	Mitochondrial PTENalpha	1.268126023	3.67E-03	up
**S100A11**	hsa:6282	Protein S100-A11	1.258993607	3.70E-03	up
**PLIN3**	hsa:10226	Perilipin-3	1.205055793	3.87E-03	up
**NFE4**	hsa:58160	Transcription factor NF-E4	1.401152119	3.98E-03	up
**AQP9**	hsa:366	cDNA FLJ50860, highly similar to Aquaporin-9	1.445829741	4.27E-03	up
**NCF4**	hsa:4689	Neutrophil cytosol factor 4	1.367265515	4.31E-03	up
**CPNE2**	hsa:221184	Copine-2	1.200804811	4.43E-03	up

**LIMK2**	hsa:3985	LIM domain kinase 2, isoform CRA_a	1.262445353	5.12E-03	up
**PLBD1**	hsa:79887	Phospholipase B-like 1	1.212475981	5.29E-03	up
**CTSE**	hsa:1510	cDNA FLJ60742, highly similar to Homo sapiens cathepsin E (CTSE), transcript variant 2, mRNA	1.23311866	5.35E-03	up
**MCEMP1**	hsa:199675	Mast cell-expressed membrane protein 1	1.619849389	5.55E-03	up
**SMIM1**	hsa:388588	Small integral membrane protein 1	1.515766933	5.57E-03	up
**HN1**	hsa:51155	Hematological and neurological expressed 1 protein	1.212294119	5.67E-03	up
**CCNJL**	hsa:79616	cDNA FLJ53725, moderately similar to Mus musculus cyclin J (Ccnj), mRNA	1.241507578	5.83E-03	up
**C9orf72**	hsa:203228	Protein C9orf72	1.255466725	6.25E-03	up
**KRT23**	hsa:25984	Keratin 23 (Histone deacetylase inducible), isoform CRA_a	1.473509177	6.61E-03	up
**LINC01410**		long intergenic non-protein coding RNA 1410	1.278290153	6.81E-03	up
**RHOG**	hsa:391	Rho-related GTP-binding protein RhoG	1.205178356	6.85E-03	up
**LOC100288781**			1.268042356	7.02E-03	up
**SULT1A2**	hsa:6799	Sulfotransferase 1A2	1.212052682	7.28E-03	up
**HCK**	hsa:3055	Non-specific protein-tyrosine kinase	1.233212067	7.47E-03	up
**MT2A**	hsa:4502	Metallothionein-2	1.203340927	8.00E-03	up
**TDRD9**	hsa:122402	Putative ATP-dependent RNA helicase TDRD9	1.458762303	8.08E-03	up
**RGL4**	hsa:266747	Ral-GDS-related protein	1.325816833	8.17E-03	up
**HK3**	hsa:3101	Hexokinase 3 (White cell), isoform CRA_b	1.243783967	8.61E-03	up
**TACSTD2**	hsa:4070	Tumor-associated calcium signal transducer 2	1.212885122	8.67E-03	up
**SERPINA1**	hsa:5265	Serpin peptidase inhibitor, clade A (Alpha-1 antiproteinase, antitrypsin), member 1, isoform CRA_a	1.221853401	8.81E-03	up
**CYSTM1**	hsa:84418	Cysteine-rich and transmembrane domain-containing protein 1	1.360726012	8.86E-03	up
**F5**	hsa:2153	cDNA FLJ50218, highly similar to Coagulation factor V	1.326022037	9.13E-03	up
**SULT1A1**	hsa:6817	Sulfotransferase 1A1	1.255078354	9.15E-03	up
**LOC100510224**			1.235515423	9.31E-03	up
**BASP1**	hsa:10409	Brain acid soluble protein 1	1.390411866	1.03E-02	up
**FCGR1B**	hsa:2210	High affinity immunoglobulin gamma Fc receptor IB	1.422121249	1.09E-02	up
**CAMP**	hsa:820	Cathelicidin antimicrobial peptide	1.279895134	1.09E-02	up
**HIP1**	hsa:3092	HIP1 protein	1.24262902	1.12E-02	up
**ABCA7**	hsa:10347	ATP-binding cassette sub-family A member 7	1.202174871	1.13E-02	up
**LMNB1**	hsa:4001	cDNA FLJ50934, highly similar to Lamin-B1	1.306522945	1.15E-02	up
**FAM20A**	hsa:54757	Family with sequence similarity 20, member A	1.206631878	1.16E-02	up

**FCGR1C**	hsa:100132417	Putative high affinity immunoglobulin gamma Fc receptor IC	1.41091827	1.22E-02	up
**ACSL1**	hsa:2180	cDNA FLJ76467, highly similar to Homo sapiens acyl-CoA synthetase long-chain family member 1 (ACSL1), mRNA	1.432799731	1.26E-02	up
**KISS1R**	hsa:84634	KiSS-1 receptor	1.260651807	1.28E-02	up
**LILRA2**	hsa:11027	Leukocyte immunoglobulin-like receptor subfamily A member 2	1.206004081	1.29E-02	up
**NFIL3**	hsa:4783	Nuclear factor, interleukin 3 regulated, isoform CRA_a	1.392136401	1.31E-02	up
**PI3**	hsa:5266	Elafin	1.441383646	1.35E-02	up
**HAUS4**	hsa:54930	HAUS augmin-like complex subunit 4	1.224043912	1.36E-02	up
**MAK**	hsa:4117	cDNA FLJ40512 fis, clone TESTI2046439, highly similar to Serine/threonine-protein kinase MAK (EC 2.7.11.22)	1.269442014	1.42E-02	up
**ST3GAL6**	hsa:10402	ST3 beta-galactoside alpha-2,3-sialyltransferase 6, isoform CRA_b	1.211035412	1.46E-02	up
**HSPA6**	hsa:3310	cDNA, FLJ93166, highly similar to Homo sapiens heat shock 70kDa protein 6 (HSP70B') (HSPA6), mRNA	1.226488852	1.47E-02	up
**FOLR3**	hsa:2352	Folate receptor gamma	1.602804527	1.49E-02	up
**FPR1**	hsa:2357	Formyl peptide receptor 1, isoform CRA_a	1.277331087	1.53E-02	up
**LOC100134822**	hsa:100134822	uncharacterized LOC100134822	1.332062605	1.58E-02	up
**GPR27**	hsa:2850	G protein-coupled receptor 27	1.201776788	1.59E-02	up
**LOC731424**	hsa:731424	uncharacterized LOC731424	1.250770364	1.62E-02	up
**MBOAT2**	hsa:129642	cDNA, FLJ79530, highly similar to Homo sapiens O-acyltransferase (membrane bound) domain containing 2 (OACT2), mRNA	1.224520498	1.65E-02	up
**STEAP4**	hsa:79689	Metalloreductase STEAP4	1.394917052	1.74E-02	up
**ZBTB24**	hsa:9841	Zinc finger and BTB domain-containing protein 24	1.283340295	1.80E-02	up
**TLR2**	hsa:7097	Toll-like receptor 2	1.263538684	1.81E-02	up
**PYGL**	hsa:5836	Alpha-1,4 glucan phosphorylase	1.318767457	1.88E-02	up
**PPP1R3B**	hsa:79660	Protein phosphatase 1 regulatory subunit 3B	1.208058052	1.90E-02	up
**BCL6**	hsa:604	B-cell CLL/lymphoma 6	1.331993921	1.93E-02	up
**DACH1**	hsa:1602	Dachshund homolog 1	1.227236557	1.94E-02	up
**CMBL**	hsa:134147	cDNA, FLJ96437	1.329937976	1.96E-02	up
**SERPINB2**	hsa:5055	cDNA, FLJ93654, highly similar to Homo sapiens serpin peptidase inhibitor, clade B (ovalbumin), member 2 (SERPINB2), mRNA	1.228647189	1.97E-02	up
**FCGR2B**	hsa:2213	Low affinity immunoglobulin gamma Fc region receptor II-b	1.284409343	1.97E-02	up
**BTNL3**	hsa:10917	Butyrophilin-like protein 3	1.213121885	2.05E-02	up
**TMEM176A**	hsa:55365	Hepatocellular carcinoma-associated antigen 112	1.333804373	2.07E-02	up

**RNASE2**	hsa:6036	Non-secretory ribonuclease	1.318803606	2.09E-02	up
**WDFY3**	hsa:23001	WD repeat and FYVE domain containing 3, isoform CRA_a	1.200853056	2.24E-02	up
**LY96**	hsa:23643	Lymphocyte antigen 96	1.288427129	2.38E-02	up
**CSRNP1**	hsa:64651	AXIN1 up-regulated 1, isoform CRA_a	1.214387141	2.42E-02	up
**LRRC6**	hsa:23639	Protein tilB homolog	1.260824745	2.46E-02	up
**VNN1**	hsa:8876	cDNA, FLJ95014, highly similar to Homo sapiens vanin 1 (VNN1), mRNA	1.372305298	2.48E-02	up
**FFAR2**	hsa:2867	Free fatty acid receptor 2	1.28933891	2.63E-02	up
**LOC646470**			1.292997233	2.67E-02	up
**NFE2**	hsa:4778	cDNA FLJ78141, highly similar to nuclear factor erythroid 2 isoform f=basic leucine zipper protein	1.216145891	2.69E-02	up
**BST1**	hsa:683	ADP-ribosyl cyclase/cyclic ADP-ribose hydrolase 2	1.261832775	2.72E-02	up
**IMPA2**	hsa:3613	Inositol monophosphatase 2	1.200104562	2.72E-02	up
**FCAR**	hsa:2204	FCAR	1.298089146	2.76E-02	up
**CD14**	hsa:929	Monocyte differentiation antigen CD14	1.258225694	2.76E-02	up
**HP**	hsa:3240	cDNA FLJ31310 fis, clone LIVER1000165, highly similar to Haptoglobin	1.438956761	2.78E-02	up
**TECPR2**	hsa:9895	Tectonin beta-propeller repeat-containing protein 2	1.204311849	2.84E-02	up
**LRG1**	hsa:116844	cDNA FLJ54228, highly similar to Leucine-rich alpha-2-glycoprotein	1.353210695	2.87E-02	up
**GNG10**	hsa:2790	Guanine nucleotide-binding protein subunit gamma	1.215037931	2.89E-02	up
**CYFIP1**	hsa:23191	Cytoplasmic FMR1-interacting protein 1	1.201900016	2.90E-02	up
**RNASE4**	hsa:6038	Ribonuclease 4	1.234515912	2.94E-02	up
**SLC22A4**	hsa:6583	Solute carrier family 22 member 4	1.322960949	3.04E-02	up
**NQO2**	hsa:4835	Ribosyldihydronicotinamide dehydrogenase [quinone]	1.267860072	3.08E-02	up
**CDA**	hsa:978	Small cytidine deaminase	1.302160866	3.13E-02	up
**MGAM**	hsa:8972	Maltase-glucoamylase (Alpha-glucosidase), isoform CRA_a	1.421724794	3.14E-02	up
**KLHL2**	hsa:11275	Kelch-like protein 2	1.268962665	3.17E-02	up
**CR1**	hsa:1378	Complement receptor type 1	1.270217917	3.27E-02	up
**NAIP**	hsa:4671	NAIP protein	1.271478661	3.29E-02	up
**CKAP4**	hsa:10970	Cytoskeleton-associated protein 4, isoform CRA_c	1.215817018	3.38E-02	up
**PLXNC1**	hsa:10154	cDNA FLJ58722, highly similar to Plexin-C1	1.283765573	3.44E-02	up
**SLC26A8**	hsa:116369	Solute carrier family 26, member 8, isoform CRA_a	1.297069644	3.46E-02	up
**ST3GAL4-AS1**	hsa:399972	ST3GAL4 antisense RNA 1 (head to head)	1.30458515	3.55E-02	up
**MMP9**	hsa:4318	cDNA FLJ51036, highly similar to Matrix metalloproteinase-9 (EC3.4.24.35)	1.537689231	3.72E-02	up

**TMEM176B**	hsa:28959	cDNA FLJ39985 fis, clone STOMA2000279, highly similar to Homo sapiens LR8 protein (LR8), mRNA	1.281079591	3.84E-02	up
**CD163**	hsa:9332	Scavenger receptor cysteine-rich type 1 protein M130	1.283543533	3.93E-02	up
**TCN1**	hsa:6947	Transcobalamin-1	1.259512017	3.96E-02	up
**CMTM2**	hsa:146225	CKLF-like MARVEL transmembrane domain-containing protein 2	1.215171655	3.97E-02	up
**GPM6A**	hsa:2823	Neuronal membrane glycoprotein M6-a	1.212468425	3.99E-02	up
**BCL2A1**	hsa:597	Bcl-2-related protein A1	1.303570594	4.00E-02	up
**PLSCR1**	hsa:5359	Phospholipid scramblase 1	1.277547617	4.08E-02	up
**KCNJ15**	hsa:3772	cDNA FLJ75829, highly similar to Homo sapiens potassium inwardly-rectifying channel, subfamily J, member 15 (KCNJ15), transcript variant 2, mRNA	1.235570993	4.14E-02	up
**VNN2**	hsa:8875	Vascular non-inflammatory molecule 2	1.294314818	4.16E-02	up
**LOC100510692**			1.271484521	4.21E-02	up
**GCA**	hsa:25801	cDNA FLJ52146, highly similar to Grancalcin	1.215366019	4.23E-02	up
**FAM129A**	hsa:116496	Chromosome 1 open reading frame 24, isoform CRA_a	1.231727455	4.25E-02	up
**SOD2**	hsa:6648	Superoxide dismutase [Mn], mitochondrial	1.202435925	4.32E-02	up
**SIGLEC5**	hsa:8778	Sialic acid-binding Ig-like lectin 5	1.241681134	4.38E-02	up
**ROPN1L**	hsa:83853	Ropporin-1-like protein	1.201454768	4.60E-02	up
**LOC100505956**			1.27198631	4.65E-02	up
**CLEC12A**	hsa:160364	C-type lectin domain family 12 member A	1.306628127	4.66E-02	up
**SLC2A3**	hsa:6515	cDNA FLJ57557, highly similar to Solute carrier family 2, facilitated glucose transporter member 3	1.209933467	4.84E-02	up
**FPR2**	hsa:2358	Formyl peptide receptor-like 1, isoform CRA_a	1.295598999	4.98E-02	up
