# Supplementary material for: Network Analysis-Based Approach for Exploring the Potential Diagnostic Biomarkers of Acute Myocardial Infarction
Source: Front Physiol. 2016 Dec 9;7:615. doi: 10.3389/fphys.2016.00615 (PMC5145872; doi:10.3389/fphys.2016.00615)
Supplement: Supplementary file 3 [file Table3.PDF]

Table 3 The relationship between GO terms in the GO-Tree

GO_ID1	GO_Term1	GOTerm1 _Style	GO_ID2	GO_Term2	GOTerm2 _Style	Relation
GO:0002523	leukocyte migration involved in inflammatory response	up	GO:0006954	inflammatory response	up	part_of
GO:0002526	acute inflammatory response	up	GO:0006954	inflammatory response	up	is_a
GO:0002544	chronic inflammatory response	up	GO:0006954	inflammatory response	up	is_a
GO:0002755	MyD88-dependent toll-like receptor signaling pathway	up	GO:0002224	toll-like receptor signaling pathway	up	is_a
GO:0006584	catecholamine metabolic process	up	GO:0018958	phenol-containing compound metabolic process	up	is_a
GO:0006953	acute-phase response	up	GO:0002526	acute inflammatory response	up	is_a
GO:0030168	platelet activation	down	GO:0007596	blood coagulation	down	part_of
GO:0032496	response to lipopolysaccharide	up	GO:0002237	response to molecule of bacterial origin	up	is_a
GO:0032602	chemokine production	up	GO:0001816	cytokine production	down	is_a
GO:0034123	positive regulation of toll-like receptor signaling pathway	up	GO:0002224	toll-like receptor signaling pathway	up	positively _regulates
GO:0045087	innate immune response	up	GO:0006955	immune response	down	is_a
GO:0050727	regulation of inflammatory response	up	GO:0006954	inflammatory response	up	regulates
GO:0050729	positive regulation of inflammatory response	up	GO:0006954	inflammatory response	up	positively _regulates
GO:0050729	positive regulation of inflammatory response	up	GO:0050727	regulation of inflammatory response	up	is_a
GO:0050830	defense response to Gram-positive bacterium	up	GO:0042742	defense response to bacterium	up	is_a
