# Supplementary material for: Network Analysis-Based Approach for Exploring the Potential Diagnostic Biomarkers of Acute Myocardial Infarction
Source: Front Physiol. 2016 Dec 9;7:615. doi: 10.3389/fphys.2016.00615 (PMC5145872; doi:10.3389/fphys.2016.00615)
Supplement: Supplementary file 4 [file Table4.PDF]

Table 4 The relationship between Pathway terms in the Pathway-Act-Network

SourcePath	Source_PathwayTerm	Source_Style	TargetPath	Target_PathwayTerm	Target_Style
PATH:04062	Chemokine signaling pathway	down	PATH:04060	Cytokine-cytokine receptor interaction	down
PATH:04062	Chemokine signaling pathway	down	PATH:04630	Jak-STAT signaling pathway	down
PATH:04140	Regulation of autophagy	down	PATH:04612	Antigen processing and presentation	down
PATH:04140	Regulation of autophagy	down	PATH:04910	Insulin signaling pathway	down
PATH:04145	Phagosome	up	PATH:04610	Complement and coagulation cascades	up
PATH:04145	Phagosome	up	PATH:04612	Antigen processing and presentation	down
PATH:04145	Phagosome	up	PATH:04620	Toll-like receptor signaling pathway	all
PATH:04540	Gap junction	down	PATH:04060	Cytokine-cytokine receptor interaction	down
PATH:04612	Antigen processing and presentation	down	PATH:04650	Natural killer cell mediated cytotoxicity	down
PATH:04620	Toll-like receptor signaling pathway	all	PATH:04060	Cytokine-cytokine receptor interaction	down
PATH:04620	Toll-like receptor signaling pathway	all	PATH:04610	Complement and coagulation cascades	up
PATH:04620	Toll-like receptor signaling pathway	all	PATH:04630	Jak-STAT signaling pathway	down
PATH:04630	Jak-STAT signaling pathway	down	PATH:04060	Cytokine-cytokine receptor interaction	down
PATH:04650	Natural killer cell mediated cytotoxicity	down	PATH:04060	Cytokine-cytokine receptor interaction	down
PATH:04650	Natural killer cell mediated cytotoxicity	down	PATH:04630	Jak-STAT signaling pathway	down
PATH:04910	Insulin signaling pathway	down	PATH:00500	Starch and sucrose metabolism	up
PATH:05130	Pathogenic Escherichia coli infection	up	PATH:04620	Toll-like receptor signaling pathway	all
PATH:05134	Legionellosis	up	PATH:04610	Complement and coagulation cascades	up
PATH:05134	Legionellosis	up	PATH:04620	Toll-like receptor signaling pathway	all
PATH:05140	Leishmaniasis	up	PATH:04145	Phagosome	up
PATH:05140	Leishmaniasis	up	PATH:04610	Complement and coagulation cascades	up
PATH:05140	Leishmaniasis	up	PATH:04620	Toll-like receptor signaling pathway	all
PATH:05140	Leishmaniasis	up	PATH:04630	Jak-STAT signaling pathway	down
PATH:05144	Malaria	down	PATH:04610	Complement and coagulation cascades	up
PATH:05144	Malaria	down	PATH:04620	Toll-like receptor signaling pathway	all
PATH:05150	Staphylococcus aureus infection	up	PATH:04610	Complement and coagulation cascades	up
PATH:05152	Tuberculosis	up	PATH:04612	Antigen processing and presentation	down
PATH:05152	Tuberculosis	up	PATH:04620	Toll-like receptor signaling pathway	all
PATH:05152	Tuberculosis	up	PATH:04630	Jak-STAT signaling pathway	down
PATH:05162	Measles	down	PATH:04620	Toll-like receptor signaling pathway	all
PATH:05162	Measles	down	PATH:04630	Jak-STAT signaling pathway	down

PATH:05164	Influenza A	down	PATH:04620	Toll-like receptor signaling pathway	all
PATH:05164	Influenza A	down	PATH:04630	Jak-STAT signaling pathway	down
PATH:05168	Herpes simplex infection	down	PATH:04610	Complement and coagulation cascades	up
PATH:05168	Herpes simplex infection	down	PATH:04612	Antigen processing and presentation	down
PATH:05168	Herpes simplex infection	down	PATH:04620	Toll-like receptor signaling pathway	all
PATH:05168	Herpes simplex infection	down	PATH:04630	Jak-STAT signaling pathway	down
PATH:05320	Autoimmune thyroid disease	down	PATH:04610	Complement and coagulation cascades	up
PATH:05320	Autoimmune thyroid disease	down	PATH:04612	Antigen processing and presentation	down
PATH:05321	Inflammatory bowel disease (IBD)	all	PATH:04060	Cytokine-cytokine receptor interaction	down
PATH:05321	Inflammatory bowel disease (IBD)	all	PATH:04612	Antigen processing and presentation	down
PATH:05321	Inflammatory bowel disease (IBD)	all	PATH:04620	Toll-like receptor signaling pathway	all
PATH:05321	Inflammatory bowel disease (IBD)	all	PATH:04630	Jak-STAT signaling pathway	down
PATH:05323	Rheumatoid arthritis	down	PATH:04620	Toll-like receptor signaling pathway	all
PATH:05330	Allograft rejection	down	PATH:04610	Complement and coagulation cascades	up
PATH:05330	Allograft rejection	down	PATH:04612	Antigen processing and presentation	down
PATH:05332	Graft-versus-host disease	down	PATH:04060	Cytokine-cytokine receptor interaction	down
PATH:05332	Graft-versus-host disease	down	PATH:04612	Antigen processing and presentation	down
PATH:05332	Graft-versus-host disease	down	PATH:04620	Toll-like receptor signaling pathway	all
PATH:05332	Graft-versus-host disease	down	PATH:04650	Natural killer cell mediated cytotoxicity	down
