# Supplementary material for: Network Analysis-Based Approach for Exploring the Potential Diagnostic Biomarkers of Acute Myocardial Infarction
Source: Front Physiol. 2016 Dec 9;7:615. doi: 10.3389/fphys.2016.00615 (PMC5145872; doi:10.3389/fphys.2016.00615)
Supplement: Supplementary file 5 [file Table5.PDF]

Table 5 The difference k-core in the co-expression network of AMI and Control group(｜Dif_Kcore｜>20)

Symbol	Description	Degree_AMI	Degree_Control	Dif_Degree	Kcore_AMI	Kcore_Control	Dif_Kcore2
CNN2	cDNA FLJ52495, highly similar to Calponin-2	48	3	45	45	3	42
CRYZ	Quinone oxidoreductase	49	6	43	42	6	36
SULT1A1	Sulfotransferase 1A1	52	12	40	45	11	34
SULT1A2	Sulfotransferase 1A2	54	14	40	45	12	33
PRMT2	Protein arginine N-methyltransferase 2	54	14	40	45	13	32
ATP1B1	ATPase, Na+/K+ transporting, beta 1 polypeptide, isoform CRA_a	49	12	37	42	12	30
FCGR1B	High affinity immunoglobulin gamma Fc receptor IB	61	18	43	45	16	29
RPL38	Ribosomal protein L38, isoform CRA_a	34	0	34	29	0	29
SMN2	Survival motor neuron protein isoform 6B	55	17	38	45	17	28
FES	Tyrosine-protein kinase Fes/Fps	103	22	81	45	19	26
HOXB2	Homeobox B2, isoform CRA_b	29	1	28	27	1	26
CD14	Monocyte differentiation antigen CD14	63	26	37	45	20	25
HIP1	HIP1 protein	54	23	31	45	20	25
ATP6V0D1	cDNA, FLJ93507, highly similar to Homo sapiens ATPase, H+ transporting, lysosomal 38kDa, V0 subunit d isoform 1 (ATP6V0D1), mRNA	62	30	32	45	22	23
FOLR3	Folate receptor gamma	24	0	24	23	0	23
LRRC6	Protein tilB homolog	34	4	30	26	4	22
C9orf72	Protein C9orf72	36	14	22	33	13	20
KIN	DNA/RNA-binding protein KIN17	45	20	25	38	18	20
MED21	SRB7 suppressor of RNA polymerase B homolog (Yeast), isoform CRA_b	32	100	-68	30	51	-21
SMAD7	cDNA FLJ16482 fis, clone BRTHA2017972, highly similar to Mothers against decapentaplegic homolog 7 (SMAD 7)	45	64	-19	24	47	-23
S100A8	Protein S100-A8	10	32	-22	7	31	-24
KLRD1	Natural killer cells antigen CD94	30	63	-33	22	47	-25
GZMA	Granzyme A	41	78	-37	24	50	-26
KLRC4	NKG2-F type II integral membrane protein	36	67	-31	24	50	-26
KLRG1	cDNA FLJ56726, highly similar to Homo sapiens killer cell lectin-like receptor subfamily G, member 1 (KLRG1), mRNA	34	74	-40	24	50	-26
SLAMF7	cDNA FLJ76627	36	73	-37	24	50	-26

TGFBR3	Transforming growth factor beta receptor type 3	38	77	-39	24	50	-26
XCL1	Lymphotactin	39	64	-25	24	50	-26
PDK4	Pyruvate dehydrogenase kinase, isoenzyme 4	23	64	-41	23	50	-27
RAP1B	RAP1B, member of RAS oncogene family, isoform CRA_a	34	84	-50	24	51	-27
KLRB1	cDNA FLJ75274, highly similar to Homo sapiens killer cell lectin-like receptor subfamily B, member 1(KLRB1), mRNA	23	63	-40	21	50	-29
CX3CR1	CX3C chemokine receptor 1	8	49	-41	8	38	-30
GCH1	GTP cyclohydrolase 1 (Dopa-responsive dystonia), isoform CRA_a	22	90	-68	20	51	-31
INSIG1	Insulin-induced gene protein	19	69	-50	19	51	-32
CXCL5	C-X-C motif chemokine 5	9	56	-47	9	43	-34
GBP3	Guanylate-binding protein 3	17	75	-58	17	51	-34
HEG1	Protein HEG homolog 1	15	89	-74	15	51	-36
