# Supplementary material for: Network Analysis-Based Approach for Exploring the Potential Diagnostic Biomarkers of Acute Myocardial Infarction
Source: Front Physiol. 2016 Dec 9;7:615. doi: 10.3389/fphys.2016.00615 (PMC5145872; doi:10.3389/fphys.2016.00615)
Supplement: Supplementary file 6 [file Table6.PDF]

Table 6 The relationship of TG-TF interactions network

TG	TG Style	TF	TF Style	zscore	type	species
SH2D1A	down	BCL11B	down	9.71	blood	Homo sapiens
SH2D1A	down	GATA3	down	9.68	blood	Homo sapiens
GZMB	down	TBX21	down	8.43	blood	Homo sapiens
GATA3	down	TXK	down	8.02	blood	Homo sapiens
PRF1	down	TBX21	down	7.66	blood	Homo sapiens
PRF1	down	BCL11B	down	7.65	blood	Homo sapiens
GATA3	down	BCL11B	down	7.64	blood	Homo sapiens
IL2RB	down	BCL11B	down	7.63	blood	Homo sapiens
IL2RB	down	GATA3	down	7.62	blood	Homo sapiens
PRF1	down	GATA3	down	7.59	blood	Homo sapiens
SOCS3	up	CSRNP1	up	7.34	all	Homo sapiens
IFNG	down	TBX21	down	7.31	blood	Homo sapiens
XCL1	down	TBX21	down	7.18	blood	Homo sapiens
KLRD1	down	STAT4	down	6.83	germ_esc	Homo sapiens
IFNG	down	TBX21	down	6.81	all	Homo sapiens
PRF1	down	TXK	down	6.73	blood	Homo sapiens
CCL5	down	TBX21	down	6.64	blood	Homo sapiens
FASLG	down	TBX21	down	6.64	blood	Homo sapiens
SH2D1A	down	TBX21	down	6.52	blood	Homo sapiens
CXCL10	down	STAT1	down	6.49	endoderm	Homo sapiens
FASLG	down	TBX21	down	6.48	all	Homo sapiens
SH2D1A	down	TXK	down	6.48	blood	Homo sapiens
TBX21	down	STAT4	down	6.47	all	Homo sapiens
IL2RB	down	TBX21	down	6.4	blood	Homo sapiens
KLRD1	down	TBX21	down	6.39	blood	Homo sapiens
IL2RB	down	STAT4	down	6.38	blood	Homo sapiens
IL2RB	down	TBX21	down	6.34	all	Homo sapiens
SH2D1A	down	STAT4	down	6.34	blood	Homo sapiens
XCL1	down	TBX21	down	6.18	all	Homo sapiens
PRF1	down	TBX21	down	6.17	all	Homo sapiens
PRF1	down	STAT4	down	6.12	all	Homo sapiens
PRF1	down	STAT4	down	6.1	blood	Homo sapiens
GZMB	down	TBX21	down	6.06	all	Homo sapiens
TBX21	down	STAT4	down	6.03	blood	Homo sapiens
SH2D1A	down	TBX21	down	5.97	all	Homo sapiens
KLRD1	down	TXK	down	5.94	blood	Homo sapiens
CXCL10	down	STAT1	down	5.9	blood	Homo sapiens
STAT4	down	TBX21	down	5.83	blood	Homo sapiens
GATA3	down	DACH1	up	5.83	non-blood	Homo sapiens
KLRD1	down	TBX21	down	5.73	all	Homo sapiens
LY96	up	STAT1	down	5.71	all	Homo sapiens
FASLG	down	GATA3	down	5.7	blood	Homo sapiens
GZMB	down	STAT4	down	5.64	all	Homo sapiens
SH2D1A	down	TXK	down	5.63	all	Homo sapiens
TLR2	up	SOD2	up	5.59	all	Homo sapiens

CCL5	down	STAT4	down	5.55	all	Homo sapiens
CCL5	down	STAT4	down	5.54	blood	Homo sapiens
SOD2	up	NFIL3	up	5.5	all	Homo sapiens
GZMB	down	GATA3	down	5.49	blood	Homo sapiens
CASP8	down	ZNF655	down	5.49	endoderm	Homo sapiens
IL2RB	down	STAT4	down	5.45	all	Homo sapiens
KLRD1	down	BCL11B	down	5.43	blood	Homo sapiens
CCL4	down	TBX21	down	5.38	all	Homo sapiens
KLRK1	down	TBX21	down	5.21	all	Homo sapiens
GZMB	down	BCL11B	down	5.2	blood	Homo sapiens
CCL4	down	TBX21	down	5.18	blood	Homo sapiens
CD14	up	SOD2	up	5.17	all	Homo sapiens
STAT4	down	BCL11B	down	5.16	blood	Homo sapiens
PPBP	down	NFE2	up	5.15	non-blood	Homo sapiens
SH2D1A	down	STAT4	down	5.13	all	Homo sapiens
STAT4	down	GATA3	down	5.13	blood	Homo sapiens
GATA3	down	BCL11B	down	5.12	all	Homo sapiens
CCL5	down	TBX21	down	5.1	all	Homo sapiens
HCK	up	RUNX3	down	5.1	endoderm	Homo sapiens
STAT4	down	TBX21	down	5.05	all	Homo sapiens
SH2D1A	down	BCL11B	down	5.03	all	Homo sapiens
GZMB	down	STAT4	down	5.03	germ_esc	Homo sapiens
CCL4	down	STAT4	down	5.01	all	Homo sapiens
KLRC3	down	TXK	down	4.96	blood	Homo sapiens
CASP8	down	ZNF655	down	4.94	all	Homo sapiens
PRKAR2B	down	NFE2	up	4.91	blood	Homo sapiens
KLRK1	down	STAT4	down	4.9	all	Homo sapiens
GATA3	down	STAT4	down	4.89	blood	Homo sapiens
GZMB	down	STAT4	down	4.88	blood	Homo sapiens
PYGL	up	TCF7L2	down	4.86	blood	Homo sapiens
FASLG	down	STAT4	down	4.81	all	Homo sapiens
FASLG	down	STAT4	down	4.81	blood	Homo sapiens
SH2D1A	down	STAT1	down	4.77	endoderm	Homo sapiens
TBX21	down	BCL11B	down	4.75	blood	Homo sapiens
CXCL10	down	SOD2	up	4.74	all	Homo sapiens
CX3CR1	down	TXK	down	4.73	blood	Homo sapiens
CCL5	down	RUNX3	down	4.73	endoderm	Homo sapiens
KLRK1	down	TBX21	down	4.71	blood	Homo sapiens
BCL6	up	NFIL3	up	4.71	endoderm	Homo sapiens
SOS1	down	SMAD4	down	4.71	non-blood	Homo sapiens
KLRC4	down	TXK	down	4.7	blood	Homo sapiens
GATA3	down	BASP1	up	4.69	blood	Homo sapiens
S1PR1	down	RUNX3	down	4.69	blood	Homo sapiens
STAT4	down	TXK	down	4.69	blood	Homo sapiens
S1PR1	down	BCL11B	down	4.67	blood	Homo sapiens
KLRD1	down	STAT4	down	4.66	all	Homo sapiens
KLRC3	down	BCL11B	down	4.65	blood	Homo sapiens

HLA-DPA1	down	RUNX3	down	4.63	endoderm	Homo sapiens
XCL1	down	GATA3	down	4.6	blood	Homo sapiens
PYGL	up	TCF7L2	down	4.57	all	Homo sapiens
KLRK1	down	BCL11B	down	4.54	blood	Homo sapiens
PRKAA1	down	SMAD4	down	4.54	blood	Homo sapiens
PTEN	up	CHD9	down	4.53	all	Homo sapiens
TNFSF4	down	DACH1	up	4.53	blood	Homo sapiens
TBX21	down	GATA3	down	4.49	blood	Homo sapiens
SH2D1A	down	BASP1	up	4.48	blood	Homo sapiens
SOD2	up	NFIL3	up	4.46	blood	Homo sapiens
GZMB	down	RUNX3	down	4.46	endoderm	Homo sapiens
CD14	up	NFIL3	up	4.42	all	Homo sapiens
HCK	up	STAT4	down	4.39	germ_esc	Homo sapiens
CCL4	down	CSRNP1	up	4.38	all	Homo sapiens
CXCL10	down	STAT1	down	4.38	all	Homo sapiens
PRF1	down	RUNX3	down	4.38	endoderm	Homo sapiens
BCL6	up	CSRNP1	up	4.36	endoderm	Homo sapiens
CX3CR1	down	BCL11B	down	4.34	blood	Homo sapiens
CCL4	down	STAT4	down	4.34	germ_esc	Homo sapiens
IFNG	down	STAT4	down	4.3	all	Homo sapiens
KLRD1	down	GATA3	down	4.29	blood	Homo sapiens
CCL4	down	SMAD7	down	4.29	blood	Homo sapiens
KLRC3	down	TBX21	down	4.29	blood	Homo sapiens
PRF1	down	TXK	down	4.28	all	Homo sapiens
CCL5	down	BCL11B	down	4.28	blood	Homo sapiens
PRF1	down	TBX21	down	4.28	endoderm	Homo sapiens
PRKAA1	down	SMAD4	down	4.27	all	Homo sapiens
PRKACB	down	MIER3	down	4.27	endoderm	Homo sapiens
KLRD1	down	STAT4	down	4.26	blood	Homo sapiens
KLRC3	down	BCL11B	down	4.24	all	Homo sapiens
S1PR1	down	STAT4	down	4.24	blood	Homo sapiens
SMAD4	down	ZNF567	down	4.24	blood	Homo sapiens
XCL1	down	STAT4	down	4.23	blood	Homo sapiens
IL2RB	down	TXK	down	4.23	blood	Homo sapiens
SOS1	down	ZNF566	down	4.22	ectoderm	Homo sapiens
SOS1	down	NR2C2	down	4.22	non-blood	Homo sapiens
PRKAR2B	down	ZNF184	down	4.21	germ_esc	Homo sapiens
CCL4	down	STAT4	down	4.2	endoderm	Homo sapiens
CASP8	down	STAT1	down	4.19	all	Homo sapiens
IFNG	down	GATA3	down	4.18	blood	Homo sapiens
GATA3	down	TBX21	down	4.18	blood	Homo sapiens
PDE3B	down	STAT1	down	4.17	blood	Homo sapiens
PRKAA1	down	ZNF83	down	4.17	blood	Homo sapiens
PPBP	down	NFE2	up	4.16	all	Homo sapiens
SOCS3	up	SOD2	up	4.16	all	Homo sapiens
SMAD4	down	ZNF451	down	4.16	blood	Homo sapiens
KLRD1	down	TBX21	down	4.15	germ_esc	Homo sapiens

IL2RB	down	BCL11B	down	4.14	all	Homo sapiens
XCL1	down	STAT4	down	4.14	all	Homo sapiens
HLA-DPA1	down	PNN	down	4.13	endoderm	Homo sapiens
KLRD1	down	ZBTB24	up	4.12	germ_esc	Homo sapiens
IL4R	up	CSRNP1	up	4.11	all	Homo sapiens
KLRC4	down	TXK	down	4.11	all	Homo sapiens
TLR2	up	STAT4	down	4.11	endoderm	Homo sapiens
SMAD4	down	ZNF83	down	4.1	blood	Homo sapiens
HSPA6	up	CSRNP1	up	4.09	all	Homo sapiens
PTEN	up	SMAD4	down	4.09	all	Homo sapiens
PRKAA1	down	ZNF304	down	4.09	blood	Homo sapiens
PTEN	up	TAF15	down	4.08	all	Homo sapiens
KLRK1	down	STAT4	down	4.08	blood	Homo sapiens
CASP8	down	RUNX3	down	4.07	all	Homo sapiens
HK3	up	PSIP1	down	4.07	blood	Homo sapiens
PTEN	up	SMAD4	down	4.07	blood	Homo sapiens
TLR2	up	BCL6	up	4.06	endoderm	Homo sapiens
PRF1	down	STAT4	down	4.03	germ_esc	Homo sapiens
KLRC3	down	GATA3	down	4.01	blood	Homo sapiens
KLRK1	down	RUNX3	down	4.01	endoderm	Homo sapiens
